# Estimation of place-based vulnerability scores for HIV viral non-suppression: an application leveraging data from a cohort of people with histories of using drugs

**DOI:** 10.1186/s12874-023-02133-x

**Published:** 2024-01-25

**Authors:** Trang Quynh Nguyen, Laken C. Roberts Lavigne, Carly Lupton Brantner, Gregory D. Kirk, Shruti H. Mehta, Sabriya L. Linton

**Affiliations:** 1 Department of Mental Health, Johns Hopkins Bloomberg School of Public Health (JHSPH), Baltimore, MD USA; 2 Department of Health Policy and Management, JHSPH, Baltimore, MD USA; 3 Department of Biostatistics, JHSPH, Baltimore, MD USA; 4 Department of Epidemiology, JHSPH, Baltimore, MD USA

**Keywords:** Vulnerability score, Place, Place characterization, Place and health, HIV, Dimension reduction

## Abstract

The relationships between place (e.g., neighborhood) and HIV are commonly investigated. As measurements of place are multivariate, most studies apply some dimension reduction, resulting in one variable (or a small number of variables), which is then used to characterize place. Typical dimension reduction methods seek to capture the most variance of the raw items, resulting in a type of summary variable we call “disadvantage score”. We propose to add a different type of summary variable, the “vulnerability score,” to the toolbox of the researchers doing place and HIV research. The vulnerability score measures how place, as known through the raw measurements, is predictive of an outcome. It captures variation in place characteristics that matters most for the particular outcome. We demonstrate the estimation and utility of place-based vulnerability scores for HIV viral non-suppression, using data with complicated clustering from a cohort of people with histories of injecting drugs.

**Supplementary Information:**

The online version contains supplementary material available at 10.1186/s12874-023-02133-x.

## Introduction

The places where people live and go about their life activities are connected to HIV-related outcomes. This connection is an active research field; a simple Google Scholar search for the combination of “neighborhood” and “HIV,” for example, results in 129,000 articles. An important part of this literature is based on the idea that place-based features of multiple types (e.g., economic, social) and levels (e.g., macro and micro) influence HIV outcomes, and should be targets of interventions -- see, e.g., the risk environment model [1]. These studies assess the links from neighborhood characteristics to HIV diagnosis, HIV care engagement and retention, and viral suppression [2–18]. Many found that adverse neighborhood-level economic and social characteristics were associated with poor outcomes [2–5, 7, 9, 10], others suggest inverse or no effect [6, 15–17], and there are some inconsistent findings across different outcomes [11–14].

In addition to being a primary exposure, there is important potential for place-based factors to play the role of third variables (confounders or moderators) in studies of *individual-level* exposures or risk factors and HIV outcomes – which constitute an important part of the HIV literature. A small number of studies have examined neighborhood factors as moderators of the association between race/ethnicity [4–6, 8] (but to our knowledge, not other individual-level variables) and HIV outcomes. It is less common that analyses of individual-level exposures adjust for place-based factors as confounders. Yet confounding by place of individual-level associations has been recognized elsewhere (e.g., the association of air pollution exposure and cognitive functioning [19]), and such confounding is theoretically justified for many individual-level exposures and HIV outcomes, as they may both be influenced by structural factors at the neighborhood or a higher level. Also, as a helpful reviewer pointed out, relationships among individual-level exposure, place-based factors and HIV outcomes may be complex with intertwined processes that unfold over time. To further advance knowledge on how HIV-related outcomes arise and inform policy and practice, it is important both to examine place as a cause and to assess the role of individual-level causes while accounting for place.

Whichever role place is conceptualized to play (exposure, confounder or moderator), an important question in any analysis is how to characterize place as an analysis variable (or variables). Measurements of place are generally multivariate, and in the current age of big data, increasingly high-dimensional. For example, a study may collect measures of economic (e.g., poverty) and social characteristics (e.g., crime), physical features (e.g., green spaces), and health-care features (e.g., distance to services), etc. at a certain neighborhood scale (e.g., census tract or ZIP code) from multiple sources. A common approach to handling such data is to apply dimension reduction techniques to obtain a single variable (or a small number of variables) that captures the most relevant information from the original measurements in some way, then use this variable in analysis. In this paper, after a brief review how this dimension reduction is typically done, we propose an additional approach that is useful when a specific outcome is of interest. We will demonstrate this approach by leveraging outcome (viral non-suppression) data from a vulnerable population – Black people living with HIV who have injected drugs in Baltimore city [20].

### Common characterization of place: disadvantage scores

Two typical dimension reduction methods are principal component analysis (PCA) [21, 22] and factor analysis (FA) [23, 24]. They both try to capture, in some sense, the most variance present in a set of raw items. PCA takes the data as points (defined by a set of *k* variables) in *k*-dimensional space and rotates the axes so that the points are most varying along the first new axis, the second most varying along the second new axis, etc. This gives an alternative representation of the data in composite variables (principal components) along these axes, which are linear combinations of the original variables, and usually only the first principal component (or the first two) is retained for use in subsequent analysis. FA takes a different approach, assuming that there are a small number of shared underlying causes (latent variables or latent factors) that give rise to the observed variables. The fitted factor model is used to predict the latent variables, which can be used in subsequent analysis. Or the factor structure is simply used to group variables, and variables within the same factor are averaged (after standardizing) to form a summary score.

Examples of PCA- and FA-based characterization of place abound. Using data from the American Community Survey (ACS), Gebrezgi et al. [25] conducted PCA on 13 ZIP code level indicators of socio-economic status (SES – including income, income disparity, poverty, education and occupation) and constructed a “neighborhood SES index” out of seven indicators, which was then examined as a predictor of retention in HIV care and viral suppression among youth in Florida. Combining ACS statistics with data from SimplyAnalytics, Sheehan et al. [15] conducted FA on 25 ZIP code level variables capturing SES, race/ethnicity, language, housing stability and homicides, and created two composite measures of neighborhood deprivation (based on 16 variables) and residential instability/crime (based on two variables); these indices were then analyzed as predictors of HIV viral suppression among men who have sex with men in a county Ryan White program. Similar PCA- and FA-based neighborhood measures have been constructed by authors studying a range of other topics outside of HIV, including maternal child health [26], cardiovascular health [27], and health care management [28].

Aside from PCA and FA, another common approach is to manually combine (by standardizing and then averaging) variables that have been carefully hand-picked to reflect the aspect of place that one wishes to measure, rather than using a statistical tool to create parsimony. This approach was used by Decuir et al. [29] and Singh [30] to create area economic deprivation indices (analyzed in association with high-risk drug injection behaviors and mortality inequalities, respectively). It was also used by Frank et al. [31] to create a neighborhood walkability index, and by Lawal & Osayomi [32] in their creation of an index of place characteristics deemed to confer social vulnerability to COVID-19 in Nigeria.

For convenience we will refer to a summary variable resulting from PCA, FA or items averaging as a “disadvantage score” (or D-score). This is often (though not always) appropriate, as most of the neighborhood indices in the HIV and health literature are about negative aspects of place that may poorly affect health. This terminology also aligns with standard practice in the literature [5, 8, 27, 29] .

Note that the construction of a D-score does not require using any outcome data – be it HIV diagnosis or viral load, preterm birth or birth weight, physical activity or heart health, or COVID-19 outcomes. In some cases, however, to demonstrate that a D-score is relevant to the research area that motivated it, some authors show that it is associated with a relevant outcome [26, 28, 31]; this is akin to assessing construct validity.

Outcome-agnostic methods have the advantage of producing summary scores that tend to be generally applicable to multiple outcomes, even if the items have been selected with a specific outcome in mind. However, a drawback is that the D-score might not capture all elements of place that is relevant to a specific outcome of interest. For example, if an important aspect of place for the specific outcome is in the fourth principal component of a PCA, a first principal component-based D-score will miss it.

## Expanding the toolbox

### Vulnerability scores based on an outcome-oriented approach

We propose to add to the researcher’s toolbox a different kind of place-based summary score, which we call the “vulnerability score” (or V-score) for a specific outcome. This is meant to serve as an additional tool, not to replace D-scores. Like D-scores, a V-score is also a function of the original place variables, but the difference is that this function is defined based on a model for a given outcome where the original place variables are predictors.

For the sake of exposition, with a set of *k* place variables, ${X}_{1},\dots ,{X}_{k}$, using the first principal component, we have a disadvantage score of the form

$${\text{D-score}}\, = \,{a_0}\, + \,{a_1}{X_1}\, + \, \ldots \, + \,{a_k}{X_k},$$

where ${a}_{1},\dots ,{a}_{k}$ are principal loadings, and the optional ${a}_{0}$ can be set to pin down a mean for the disadvantage score. With a continuous outcome $Y$, if we use the simplest prediction model, linear regression, we obtain a vulnerability score that is the linear predictor from the fitted model,

$${\text{V-score}}\, = \,{b_0}\, + \,{b_1}{X_1}\, + \, \ldots \, + \,{b_k}{X_k}.$$

While these two scores have the same functional form, the coefficients are different, and the V-score is more predictive of *Y* than the D-score. Both are summary scores of place characteristics; the V-score is a summary that is more relevant to the outcome *Y*. Conceptually, a V-score can be used in similar ways to a D-score, e.g., to characterize place in subsequent analyses looking at place as a risk factor, or to control for place while examining the role of individual level factors.

A convenience of the V-score approach is that it is not restricted to a linear model but can benefit from flexible modeling (via machine learning). This allows searching for a flexible function,

$${\text{V-score}}\, = \,\beta ({X_1}, \ldots ,{X_k})$$

of the place measurements that is most predictive of the outcome. It thus can draw from knowledge that has been gained and tools that have been developed for predictive modeling from other contexts. We refer the interested reader to [33] for an accessible introduction to machine learning, and [34] for a textbook treatment of prediction models.

Regarding interpretation, it should be clarified upfront what the V-score is and what it is not. The V-score is a function of the place variables ($X$), not of the outcome. It is also not an estimate of the outcome for a place. What it is is the expectation of the outcome for any place with such values of the $X$ variables. Different places with the same $X$ values share the same V-score, but likely vary in the outcome because there are many causes of the outcome beyond the $X$ variables. The V-score simply indicates vulnerability to the outcome based on the place characteristics being considered.

### A case of V-score estimation and examples of V-score use

In this paper we demonstrate the estimation of V-score for HIV viral non-suppression based on census tract-level data on crime/policing activities and economic and housing conditions. The data used includes place-based variables for a range of years, and individual-level outcome data from a longitudinal study that aligns with the same years of place-based variables. This data structure is complicated due to several types of clustering. Our goal here is to showcase how we address these clustering issues.

Many tools can be used to fit flexible models (e.g., generalized linear models with splines, generalized additive models, random forests, neural nets), all of which can generally be used for V-score estimation. For our data, we need a tool that can handle clustering. We choose to use random forests [35], a tree-based method for fitting flexible models that handles nonlinearity well. More specifically we use an implementation of random forests that handles clustering [36].

A challenge with the use of outcome data in estimating V-scores (and to establish construct validity of D-scores) is that this results in the outcome data being in a sense “spent” and thus no longer suitable for use in subsequent analyses that use the V-score to characterize place. We craft a careful way to minimize this issue using “out-of-bag” prediction (explained later) so that outcome data in a place (a census tract) are completely not involved in the computing of the V-score for the place. A secondary motivation to demonstrate V-score estimation using random forests is that out-of-bag prediction is a built-in operation with random forests. While our data requires a manual solution, in simpler settings this built-in option suffices.

We will also illustrate how the estimated V-score is used in subsequent analyses (i) examining the association of living in a census tract with higher versus lower V-score and HIV viral non-suppression one year later, adjusting for individual-level covariates; and (ii) examining the association of current injection drug use with the same outcome, adjusting for individual-level covariates and census tract-level V-score.

## Data

### Place data

The place units are 200 census tracts (with boundaries defined according to the 2010 Census) of Baltimore city. The census tract data we use include variables compiled by the Baltimore Neighborhoods Indicators Alliance and extracted from the US Census Bureau American Community Survey (ACS). They include crime/policing event counts tracked by Baltimore City Police Department (seven variables); housing conditions tracked by the Baltimore City Department of Housing and other agencies (five variables), and economic conditions tracked by ACS (seven variables) – see Table 1. We restrict the analysis to the period 2009–2016 when most census tract variables and outcome data are available.

**Table 1:** Place variables

Variable	Description
**Crime/policing (rates per 1000 residents) – from Baltimore City Police Department**	
part1	Part 1 crimes (including homicide, rape, aggravated assault, robbery, burglary, larceny, auto theft)
violent	violent crimes (including homicide, rape, aggravated assault, other robbery)
gunhom^a^	homicides by firearm
narcotic	drug market activity (measured in emergency calls for narcotic-related offenses)
shoot	emergency calls for shootings
domvio^b^	emergency calls for domestic violence
juvedrug^c^	arrests of juvenile persons for possession, sale, manufacture or abuse of illegal drugs
**Housing conditions – numerator from Baltimore City Department of Housing (unless otherwise stated) / denominator from US Census American Community Survey**	
foreclose	percentage of housing units receiving foreclosure filing (from Maryland Judiciary Case Search System, MJCSS)
ownoccupy^d^	percentage of housing units that are owner-occupied (from MJCSS)
renovate	percentage of housing units with permits to renovate at $5K+
vacant	percentage of housing units that are vacant or abandoned
codeviol	percentage of housing units with housing code violations
**Economic deprivation – from US Census American Community Survey (ACS)** ^e^	
lowwage	percentage of employed persons aged 16 + in low wage occupations
poverty	percentage of individuals whose income in the past 12 months is below the poverty line
femalehh	percentage of households that are female-headed households with children under 18
pubassist	percentage of households on public assistance
nohschool	percentage of adults aged 25 + without high school diploma/GED
unemploy	percentage of persons 16 + in labor force who are unemployed
nocar	percentage of occupied housing units that do not have a vehicle

^a^ not available for 2009

^b^ available only for 2009–2011

^c^ not available for 2016

^d^ not available for 2013 and 2015

^e^ These variables result from aggregation of ACS data over a five-year interval ending in the year of interest

The place data come at the level of place-by-time, specifically census tract by year. We aim to first estimate a V-score at this level (with a value for each census tract for each year), and then collapse the years to create a version that is not year-specific. Both versions may be useful depending on the subsequent analyses that use them.

More information about the distributions of place variables are shown in Appendix A (in the supplemental material). The crime/policing variables are highly skewed and with a lot of zeros. The housing and economic deprivation variables are proportions bounded by 0 and 1. The crime/policing variables are positively correlated with one another, as are the economic deprivation variables. The housing variables have correlations with mixed signs. As mentioned above, while these features of the data would require close attention for D-score procedures, they are not of concern for V-score estimation where we will use a flexible modeling tool.

### Outcome and target population

HIV viral non-suppression is the outcome of interest. We investigate this outcome among Black people who have ever injected drugs (PWID) and are living with HIV in Baltimore city – a predominantly Black city where injection drug use is prevalent. This is a population that is disproportionately burdened by HIV [16, 37, 38], and may be particularly vulnerable to adverse neighborhood conditions due to the manner in which structural racism and stigmatization of drug use intersect [15, 17, 39, 40].

### Outcome data

Before describing the outcome data used, for conceptual clarity it helps to consider what would be the ideal. The ideal outcome data for V-score estimation would be the outcome prevalence (i.e., prevalence of HIV viral non-suppression among Black PWID living with HIV) in each census tract for each year. With such data we would fit a model for the prevalence with place characteristics as predictors and compute the V-score as the model-predicted prevalence given place characteristics. Yet prevalence at such a level of place-by-time resolution is unknown in most settings and for most outcomes.

The second ideal would be to have, for each place-by-time (here census tract by year) cell, a random sample from the target population – with similar cell-specific sample sizes. Intuitively, with such data we could use a two-step method of first obtaining estimates of outcome prevalence for each cell and then using them to fit the model that estimates the V-score. Or we could use a one-step method of regressing the outcome on cell-specific place variables (appropriately handling clustering) and computing the V-score based on that model. Like the first ideal, this second ideal is not available for the current application, and unrealistic generally.

Instead, we will make the best use of a source of HIV non-suppression data we have access to, the AIDS Linked to the IntraVenous Experience study (ALIVE) [20]. Located in Baltimore city, ALIVE is the longest-running community-based cohort of people with histories of injecting drugs in the United States. The cohort began with 2,921 adults recruited between 1988 and 1989 through community street outreach. Participants were eligible if they were ≥ 18 years, reported injection drug use within the past 11 years, and if HIV positive, were not diagnosed with AIDS at enrollment. To make up for deaths and losses to follow up (about 7%/year), five recruitment cycles (1994–1995, 1998, 2000, 2005–2008, 2015–2016) were implemented using similar eligibility criteria. Participants living with HIV attend semiannual visits, during which blood draws are taken for laboratory tests, including HIV RNA quantification via ultra-sensitive assay. Participants give their addresses at each visit, which have been linked to census tracts. The data we use are restricted to those from self-identified Black participants who were living with HIV, reported at least one visit with an HIV care provider, and lived in a residential census tract in Baltimore city during the period of interest, 2009–2017. We specifically restricted to Black participants because over 90% of ALIVE participants living with HIV are Black, so prediction for other race groups would be unreliable given limited data. Observations prior to seroconversion and prior to the first reported use of highly active antiretroviral therapy were dropped. Viral non-suppression is defined as having a viral load of 400 + copies.

**Fig. 1:**
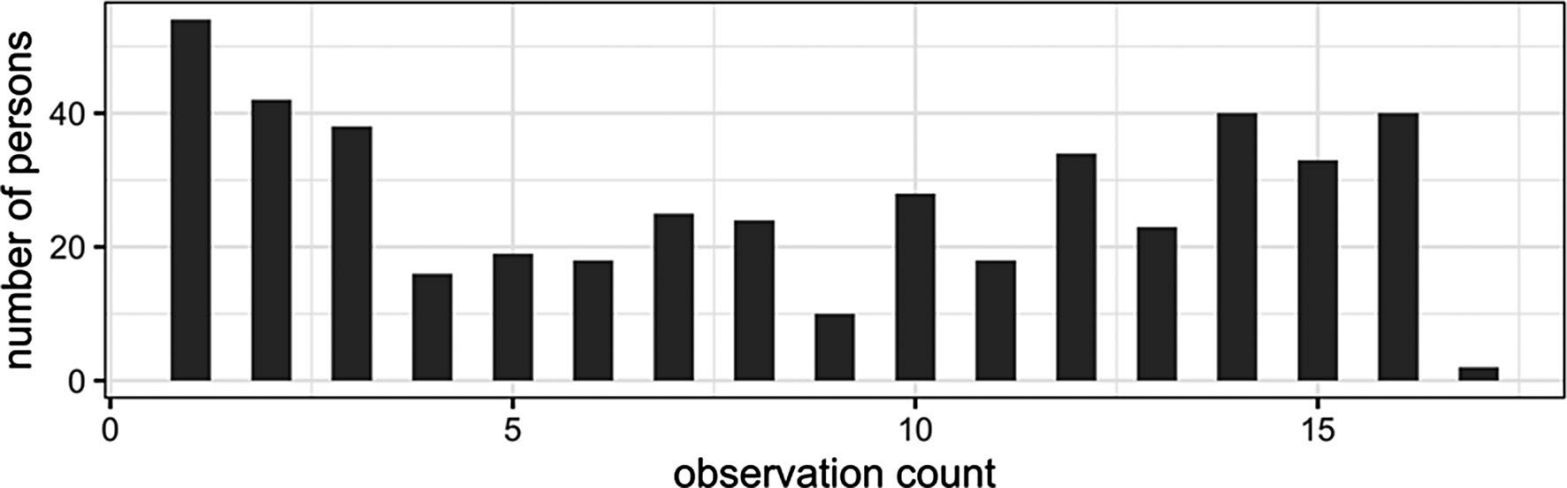
Histogram of number of observations per individual in the raw dataset

**Fig. 2:**
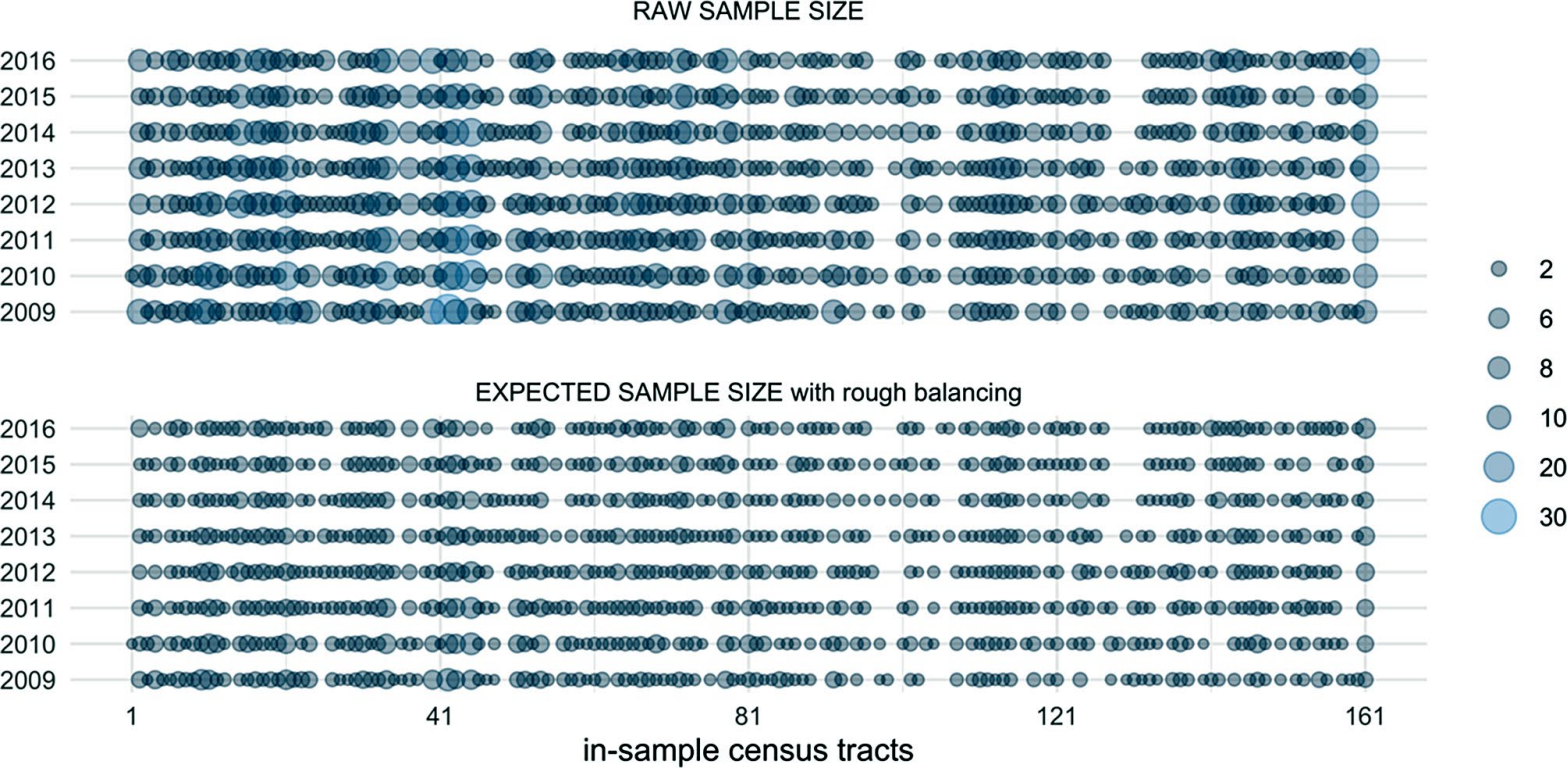
Dot plots of sample sizes of place-by-time cells: raw sample sizes (top panel, total 3890) and expected sample sizes with rough balancing (bottom panel, total 1167)

The outcome data we have is thus a sample that is followed up and replenished over time. It includes 464 individuals who provided a total of 3890 observations, which fall in 161 of the 200 residential census tracts for which we have place data, and cover 957 place-by-time cells. The number of observations contributed per individual ranges from 1 to 17 (see Fig. 1), and the place-by-time cell sample sizes range from 0 to 30 (Fig. 2, top panel). This is a complicated situation of unbalanced data with clustering of several types. There is also a trend of decreasing viral non-suppression over time. We will discuss these issues one by one and propose some strategies for resolution or mitigation.

## V-score estimation

To remind of the big picture, we want to fit a model for the outcome (HIV viral nonsuppression) using place characteristics and time as predictors, and then use the model to compute predicted outcome for each place at each time. This prediction is at the place-by-time cell level because the predictor variables are at this level and not the individual level. There are a lot of subtleties about the fitting of such a model and about how to construct the V-score based on outcome predictions. We will consider those issues after a pause to introduce random forests.

### A brief introduction to random forests

As mentioned earlier, a strength of the V-score approach is that it can benefit from flexible modeling tools that have been developed for prediction. Many machine learning algorithms can be used for this purpose. Here we use random forests [35]. Random forests are extensions upon regression trees [41]. Roughly speaking, a regression tree fits a flexible regression model by recursively partitioning the covariate space, i.e., making splits on covariates where each split is chosen (among all potential splits) to maximize the difference in outcome mean between the two sides of the split. This is called growing the tree, where each non-terminal split creates branches, and the terminal splits define the leaves of the tree. Once the tree is grown, the regression function is obtained by taking the mean of the outcome within the leaves. A random forest is the collection of a large number of regression trees, each fit to a randomly selected subsample rather than to the full sample, and each using a random subset of the covariates. A random forest is used for prediction via averaging the predictions of the trees. Intuitively, trees are noisy fits, so averaging them provides a smoother fit and thus better prediction; and data and covariates are randomly subset to reduce the correlation among the trees. Many software packages have been developed to fit random forests offering different features for parameter tuning, missing data methods, handling clustered data and accommodating big data, etc. In the R language well-known packages include ranger [42], party [43], randomForest [44] and grf [36], to name a few.

When a random forest is used for prediction on a new data point (not part of the original sample), that is called out-of-sample (OOS) prediction. This is done by averaging the predictions by all the trees in the forest. Prediction for a data point in the original sample, on the other hand, is not done by averaging all the trees, as the trees that have seen that data point may overfit that point. Instead, prediction for an in-sample data point is done by averaging all the trees fit to subsamples that did not include that data point; this is out-of-bag (OOB) prediction. With OOB prediction, the outcome of a data point is not used in the computation of the prediction. This is helpful, as it allows use of the outcome data in subsequent analyses.

Aside from the high-level themes of flexible modeling and OOS/OOB prediction, the specifics of V-score estimation depend on the data. We now address five challenges posed by our data source, which may also be present elsewhere. Readers are welcomed to focus on the challenges that are relevant to their applications and skip those that are not. For ease of reference, Table 2 summarizes the issues and the gist of the strategies used to handle them.

**Table 2:** Method issues in the application and strategies for resolving or mitigating them

Method issues	Resolution/mitigation strategies
1. The place characteristics data is clustered in place units.	Use random forest procedure that handles clustering, specifically when drawing subsamples to grow trees and when computing out-of-bag (OOB) predictions.
2. Places are connected by individuals.	Manual leave-one-out (LOO) procedure where prediction for each census tract is based on a model fit to data where not only that census tract is excluded but also all persons ever seen in that census tract are excluded.
3. Outcome data is clustered in individuals and unbalanced.	“Rough balancing”: sample a maximum of 3 time points (years) per individual and 1 place per individual-year.
4. Some place variables are not available for all years.	Attempt using as proxy the adjacent year version of the variable. If mean square error is not improved, revert back.
5. There are time trends in the data.	Remove time trends by standardizing the predictions for the census tracts within each year (to mean 0, variance 1), and use this as the V-score.

### Method issue 1: the place characteristics data is clustered in place units

When place data spans multiple time points (here years), such data is clustered in place units. We thus wish to use a modeling tool that handles clustering. Among the many software options for random forests, we use the R package grf [36] because it accommodates clustering. This package handles clustering properly both (a) when drawing subsamples to grow trees (clusters are sampled first and then units are sampled within clusters) and (b) when computing predictions (OOB prediction uses trees that do not include the cluster being predicted for). (a) ensures that the resulting model is not too confident due to wrongly treating the observations within clusters as independent (mistaking that there is more information than there actually is). (b) ensures that outcome data from a cluster is not involved in predicting for the cluster.

If the outcome data comes from repeated surveys (not a cohort as in our current application), clustering in place units is the only issue, and one could fit a single random forest, use OOB prediction for place-time units present in the sample and OOS prediction for place-time units outside the sample. For code, see Appendix A.

### Method issue 2: places are connected by individuals

In our illustrative example, the outcome data is not from repeated surveys but from a cohort of individuals, who may be seen in multiple places at different time points.

The key point of OOB prediction is that the prediction on a unit is based only on the values of its regressors and does not touch its outcome variable. The OOB prediction from the clustered random forest above ensures that the prediction for census tract A is not based on outcome data seen in census tract A. In our current example, however, some individuals may have lived in both census tracts A and B, so their outcome data in the two census tracts are connected. Therefore, we want to exclude from the building of the model used to predict for census tract A not only all outcome data seen in census tract A, but also all outcome data of such a census-tract-crossing individual that is seen elsewhere.

To achieve this, instead of simply using OOB prediction from a single random forest as mentioned above, we manually implement a leave-one-out (LOO) procedure where for one census tract at a time, we remove data of that census tract and data from all persons connected to that census tract, fit a random forest to the remaining data and use it to predict for the census tract that has been left out. The code for this method (see Appendix A) is slightly more complicated and would take longer to run, as it requires fitting one LOO random forest for each of the 161 census tracts that are in the sample. For the 39 out-of-sample census tracts, we would compute outcome predictions based on a random forest fit to the whole sample.

### Method issue 3: outcome data is clustered in individuals and unbalanced

This feature of the data causes two problems: individuals who were seen for many visits overall have more influence than those with few visits (see Fig. 1); and individuals who stayed in the same census tract (or who move around but within census tracts that have similar characteristics) have high influence on that (those) census tract(s) and may skew the results.

As a first step to reduce the problem of varying influence, within each place-time cell, we downweight individuals that appear more than once so each person has a count of at most 1 in each cell. This pares down some of the large individual weights.

It remains, however, that some individuals are present in the sample for all time points, and for up to 14 place-time cells, while others are present for only one or two points. The cleanest way to deal with this problem would be to sample one data point for each individual and discard all the rest. This would completely remove the dependence of outcome data within individuals, which is desirable. However, it would drastically reduce sample size and throw away a lot of data, which is not desirable.

We make a less extreme choice, which we call “rough balancing”: to sample a maximum of three time points (i.e., years) for each individual, and sample one place per year (if the individual reported residing in more than one census tracts in a year). This allows each individual to contribute a maximum of three data points. Our motivation in making this choice is to make the data more balanced and reduce the disproportionate influence of long-run participants. We accept the remaining dependence in the data to prevent too large a sample loss (the resulting sample, n = 1167, is 30% of the naive sample size).

As this is random sampling, to stabilize estimates, we do this multiple times and average the predicted values. We implement ten repetitions. Each repetition includes one LOO random forest for prediction on each of the in-sample census tracts, and one non-LOO random forest for prediction on the out-of-sample census tracts (see code in Appendix A). Since the repetitions are separate, they can be parallelized to speed up computing, or they can be run one at a time and results from each run can be saved to clear memory.

The expected sample of these random repetitions is represented in the bottom panel of Fig. 2. Compared to the raw place-time cell sizes of 0 to 30, the effective cell sizes are substantially smaller, 0 to 8.

A note on parameter tuning: The algorithm that fits random forests uses hyper parameters that govern tree-growing behavior (number of trees, sampling fraction, split balance tolerance and minimum node size) and for the grf package also *honesty* behavior. (Honesty uses one subsample to grow a tree and then another subsample to compute leaf values, whereas conventionally the same subsample is used for both.) These parameters have default values, but tuning them may improve performance. As our LOO random forests are all part of the implementation of a single (albeit complicated) procedure, we want to fit them using the exact same algorithm. Manual parameter tuning using several of these forests does not converge to a stable parameter set, however, so we mostly revert to default values. We opt to use honesty and increase sampling fraction to 0.7 (from the 0.5 default) based on the fact that these give the non-LOO forest good test calibration (see documentation of the related function).

**Fig. 3:**
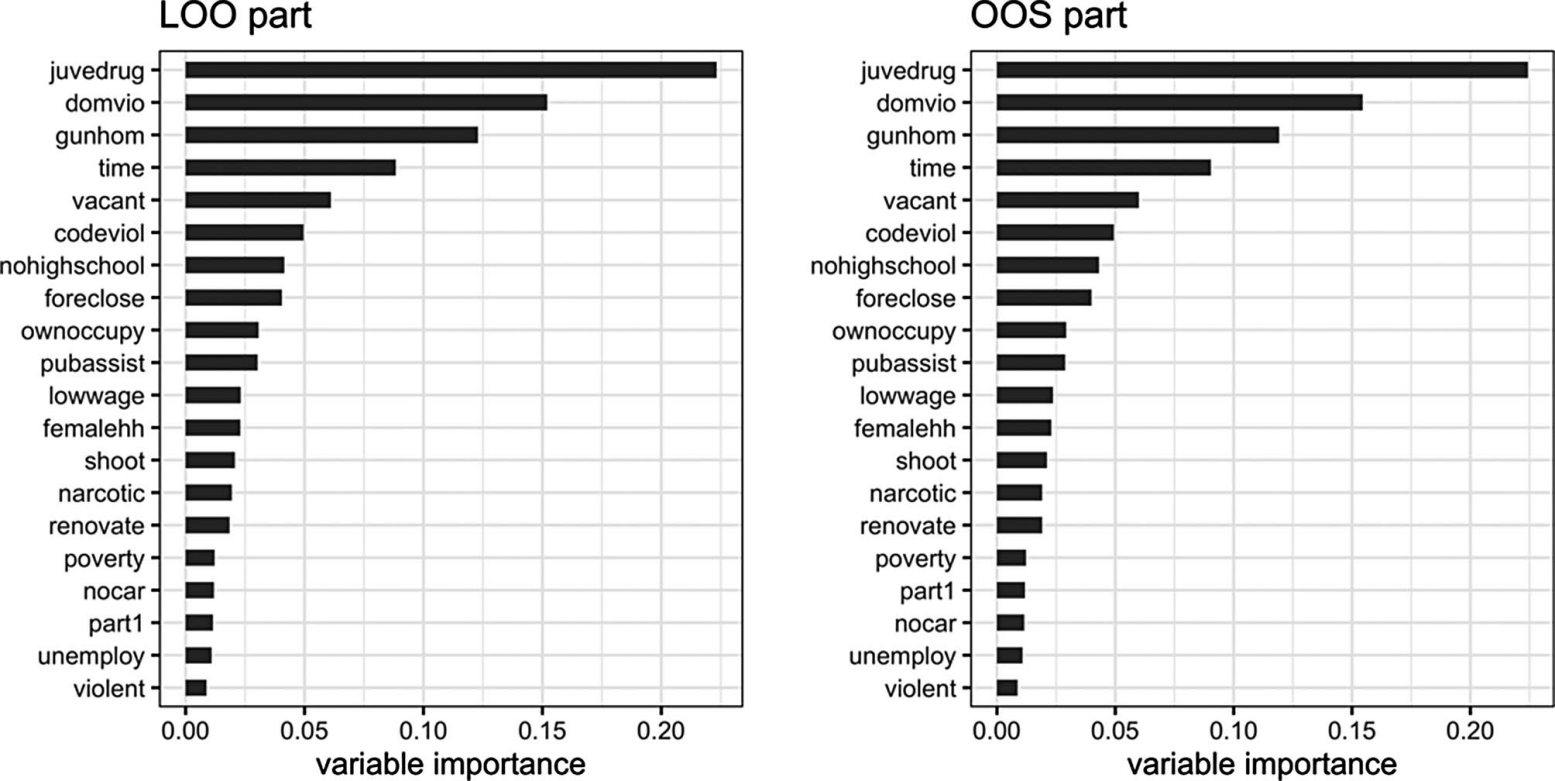
Average variable importance of LOO (left) and non-LOO (right) random forests

After running this procedure, we examine the variable importance metric to see which variables are most important for predicting the outcome. The right panel of Fig. 3 is the variable importance plot based on the single non-LOO random forest used to predict for out-of-sample census tracts, averaged over the ten repetitions; the left panel shows the average of variable importance over the 161 LOO random forests over the ten repetitions. The three top predictors are crime/policing variables *juvedrug*, *domio* and *gunhom*, followed by *time* (year).

**Fig. 4:**
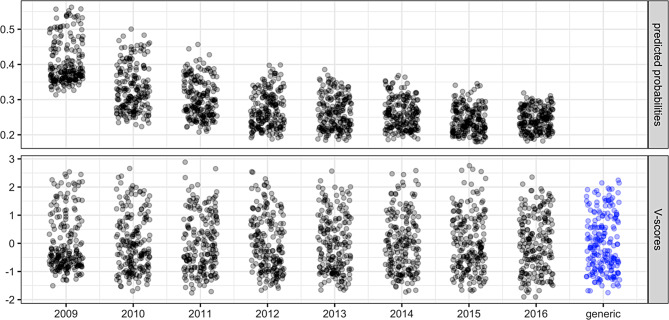
Raw predictions (top panel) which reflect time trends, year-specific V-scores (bottom panel, black) standardized to remove time trends, and generic V-scores (bottom panel, blue) combining all years. Each point represents one of Baltimore’s 200 census tracts. Points are jittered horizontally for visibility

The predictions (probabilities of viral non-suppression given place charactersticis) from this procedure are shown in the top panel of Fig. 4.

### Method issue 4: some place variables are not available for all years

While restricting analysis to 2009–2016 greatly standardizes data availability, some variables are still not available for all the years in this range. Of the three place variables with the largest variable importance, *domvio* is available for only three of the eights years, so V-score estimation for those years benefit from more data. The other two variables, *juvedrug* and *gunhom*, each are available for all but one year – 2016 for *juvedrug* and 2009 for *gunhom*. A question is whether we can do better for these two years. A reasonable idea is to bring in a proxy for the unavailable variable if such a proxy variable is available. We need to be cautious about the use of proxy variables, though, as they bring both relevant information and noise. In the current case, the variable that might serve as proxy is the same variable from an adjacent year. This would introduce another layer of complexity, since the V-score estimated would have slightly different meaning depending on the year, which may complicate/limit its utility for users.

That said, we tried this ad hoc strategy. We ran the whole procedure described above twice, each time replacing one of the two variables *juvedrug* and *gunhom* with its adjacent year version. We then take the (raw) scores for 2016 from the first ad hoc procedure and for 2009 from the second ad hoc procedure to replace the scores for those years from our original results. This increased the variability of the predicted outcome probabilities for 2016 and 2009, but slightly worsened mean squared error. We thus revert to the results from the previous step and discard this ad hoc version.

### Method issue 5: time trends in the outcome data

As described earlier, ALIVE is a complicated sample in several ways. Putting aside the fact that observations are clustered in individuals (which we partially dealt with above), this being a cohort means that there is a tendency for observations in a subsequent year to be one year older and one year later in HIV-positive life than those in the previous year; this is a continuous process as people age continuously. To some degree this is offset in a discontinuous way by the later recruitment waves. This means there may be different time trends in the data simply due to the aging of the participants and the multiple recruitment waves. In addition, there may be contextual changes of treatment becoming more accessible and/or effective over time.

What we observe is that in the sample (after rough balancing) the raw prevalence of viral non-suppression decreased from 53.1% in 2009 to 21.7% in 2016. This decreasing viral non-suppression trend is also reflected in the predicted probabilities (Fig. 4, top panel), where the average over the census tracts decreased from 40.6% in 2009 to 24.3% in 2016.

Note that our goal is to obtain a V-score that is a function of just place characteristics (which may vary with time), but not of time on top of place characteristics. (Any analyses that use the V-score can handle the effect of time by including a time variable.) To remove time trends (either general trends or trends due to the idiosyncracies of the sample), we standardize the predicted probabilities (for the 200 census tracts) within each year, so they all have mean 0 and variance 1. This obtains the year-specific V-scores shown in black color in the bottom panel of Fig. 4.

### Last consideration: year-specific and generic V-scores

While there is variation in V-scores across years – just like there is fluctuation in place characteristics – the pattern of how the census tracts compare seems persistent over the years (see Appendix A), and the V-scores estimated for the different years are highly correlated (Pearson correlations ranging from an average of 0.91 for adjacent years to 0.77 for a seven-year lag). We additionally compute a set of *generic* V-scores that combine the scores across the eight years, shown in blue in the bottom panel of Fig. 4. This set could be interpreted approximately as a general feature of the census tracts.

Both versions of the estimated V-scores may be available from the authors upon request.

## Illustration of V-score use

**Fig. 5:**
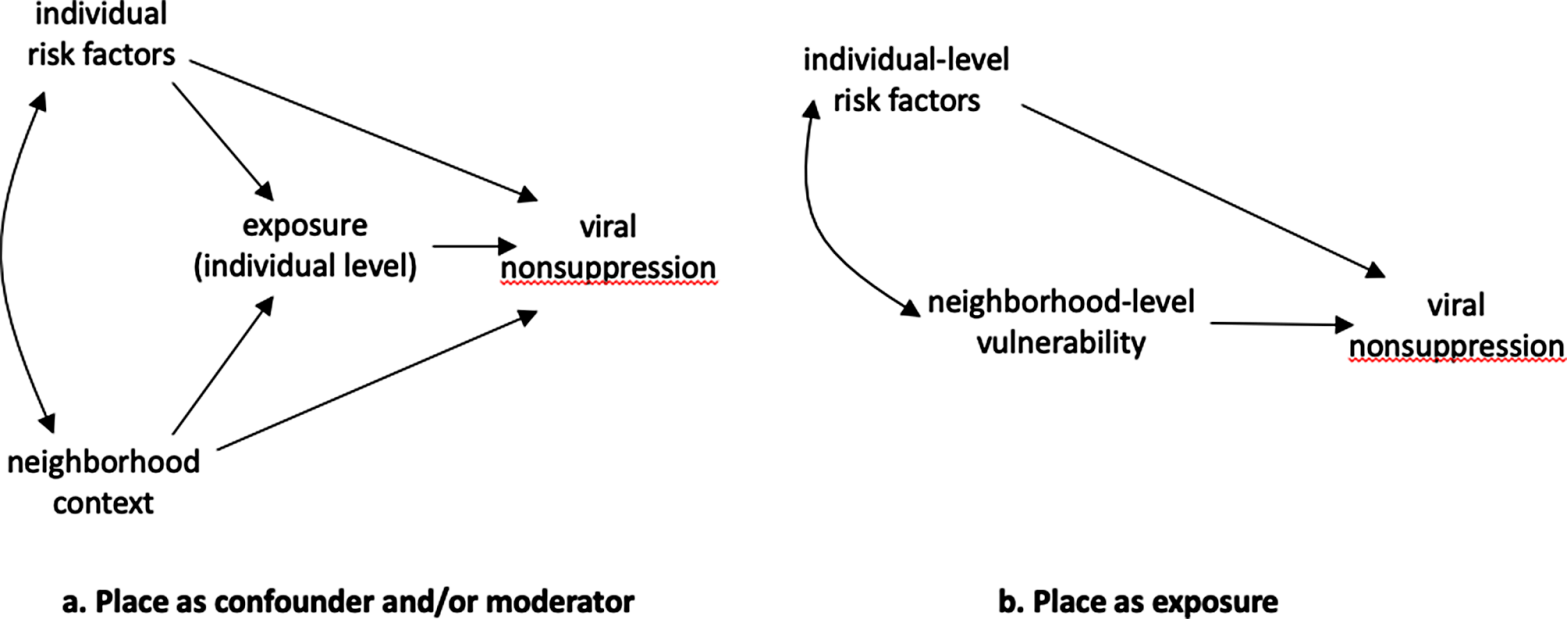
Two examples of conceptualization of role of place vis-à-vis HIV outcomes

To illustrate the utility of the V-score, we demonstrate analyses that treat the V-score either as a covariate to be adjusted (i.e., confounder) or as exposure (see Fig. 5 for relevant causal diagrams). R-code and computing outputs are in Appendix B (in the supplemental material). For convenience, these analyses use individual-level data also from the ALIVE cohort. The estimated V-scores, however, can be used in combination with other HIV-related samples from Baltimore. Also for simplicity, all these analyses use the generic version of the V-score.

These analyses are purely illustrative; results should not be interpreted as substantive findings. The intention is to demonstrate the utility of the V-score in some familiar types of analyses. Readers who find the usefulness of the V-score obvious are welcome to skip this section.

### Example analysis 1: V-score as a confounder

This first analysis examines, in the ALIVE sample of people with drug injection history, whether (i) current injection drug use (IDU) is associated with (ii) being virally nonsuppressed a year later, after adjusting for both individual-level covariates and relevant neighborhood context that may confound the relationship. In this analysis, current IDU is approximately captured by the binary variable “any IDU in the past 6 months”. Note that the focus here is not on IDU per se; this is just an example for an individual-level exposure variable.

To keep the example simple, we sample for each individual in the ALIVE study two visits about one year (10–14 months) apart, where at the first visit (aka baseline) the person reported current IDU or not, and at the second visit (follow-up) the person had a recorded viral load. We restrict the first visit to be one that falls in 2009–2016, the period for which we had estimated V-scores. This results in a sample of 405 persons, 110 (27.2%) of whom engaged in IDU at baseline. Individual-level baseline covariates include age, sex, several prognostic variables (viral suppression status, viral load and CD4 count) and several risk factors (low income, health insurance, depression, jail, homelessness, number of moves in the past 6 months) – see variable descriptions and summary statistics in Table 3. Importantly, this analysis also adjusts for the neighborhood V-score; the rationale is that neighborhoods with higher V-scores tend to embody structural factors that increase vulnerability to persistent IDU or relapse and that may also poorly affect HIV outcomes.

**Table 3:** Baseline covariates in the sample for analysis 1 – with V-score as a covariate

	Not current IDU (n = 295)		Current IDU (n = 110)	
	*mean*	*(SD)*	*mean*	*(SD)*
Age	53.0	(6.56)	51.3	(6.52)
Sex	*number*	*(percent* ^*a*^ *)*	*number*	*(percent* ^*a*^ *)*
male	198	(67.1)	78	(70.9)
female	97	(32.9)	32	(29.1)
On antiretroviral treatment				
yes	235	(79.7)	79	(71.8)
no	53	(18.0)	30	(39.1)
Viral non-suppression				
yes	112	(38.0)	65	(59.1)
no	180	(61.0)	43	(27.3)
	*max*		*max*	
Viral load	930 K		702 K	
	*mean*	*(SD)*	*mean*	*(SD)*
CD4 count	450	(296)	344	(242)
Annual income < $5K	*number*	*(percent* ^*a*^ *)*	*number*	*(percent* ^*a*^ *)*
yes	198	(67.1)	80	(72.7)
no	93	(31.5)	28	(25.5)
Health insurance^b^				
yes	286	(96.9)	103	(93.6)
no	9	(3.1)	6	(5.5)
Elevated depressive symptoms^c^				
yes	54	(18.3)	32	(29.1)
no	241	(81.7)	78	(70.9)
Any time in jail^b^				
yes	10	(3.4)	11	(10.0)
no	261	(88.5)	92	(83.6)
Any time homeless^b^				
yes	18	(6.1)	15	(13.6)
no	276	(93.6)	94	(85.5)
Number of moves^b^				
0	240	(81.4)	80	(72.7)
1	28	(9.5)	14	(12.7)
2	27	(9.2)	16	(14.5)
	*mean*	*(SD)*	*mean*	*(SD)*
Neighborhood V-score	0.43	(0.89)	0.63	(0.89)

SD = standard deviation

^a^Proportions may not add up to 100% due to missing data

^b^These variables are about the past 6 months

^c^Defined as scoring 23 + on the CES-D scale [45] for the past week

We handle missingness in the covariates (including V-score) by multiple imputation (MI) by chained equations (using the mice package [46]), creating 50 imputed datasets. To combine MI and propensity score analysis, we use what is commonly known as the *within* method, where propensity score weighting is conducted in each imputed dataset before pooling estimates by Rubin’s rules [47]; this method has been proved to be consistent [48]. In this case, the combination of V-score and individual prognostic variables is hard to balance, so we estimate propensity score weights by generalized boosted models (using the gbm package [49]), which obtain better balance than weights based on logistic regression. The whole procedure on imputed data is run using the MatchThem package [50].

**Fig. 6:**
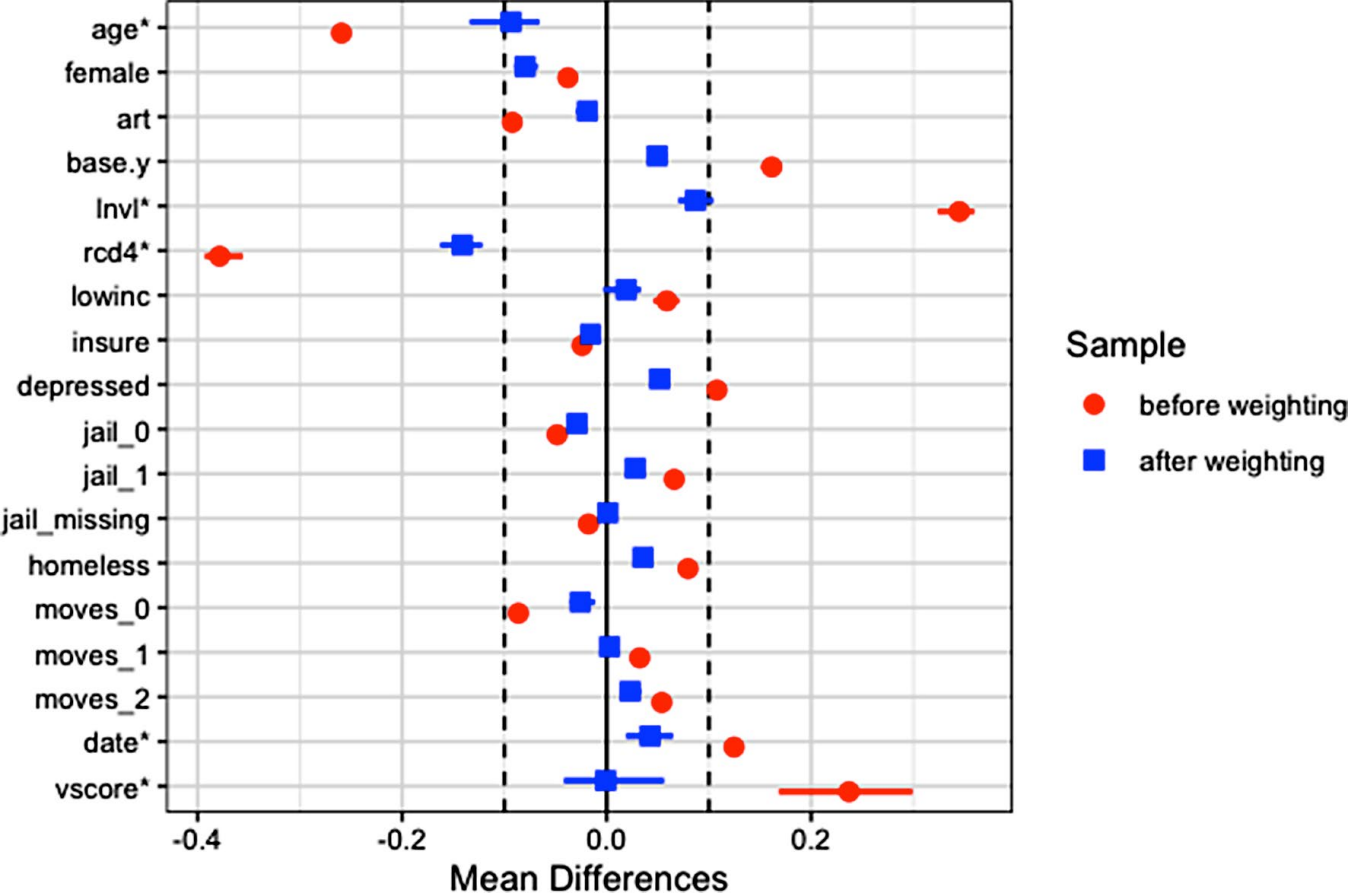
Analysis 1 – Covariate balance before and after weighting between current IDU and not current IDU groups. The horizontal bars represent variation across imputed datasets. Mean differences for continuous variables (marked with *) are standardized

Figure 6 shows that the weighting substantially improves covariate balance, including balance on the V-score. With the binary outcome, we estimate the association of interest on both the risk difference (RD) and conditional odds ratio (OR) scales. On the RD scale, after adjusting for individual-level covariates and neighborhood V-score, current IDU is associated with an RD for viral non-suppression one year later of 7.8 percetage points, 95% confidence interval (CI) = (0.6, 15.0), p-value = 0.03. On the conditional OR scale, current IDU is associated with an OR for viral non-suppression one year later of 2.02, 95% CI = (1.13, 3.59), p-value = 0.02.

Notably, the adjustment for neighborhood V-score seems warranted, as on average neighborhood V-score is higher for persons with than for persons without current IDU (see Table 3; Fig. 5). Not adjusting for V-score would result in a larger RD (8.8 percentage points, 95% CI = (1.5, 16.1), p-value = 0.02), an overestimation of the relationship of interest.

### Example analyses 2 and 3: V-score as exposure

The general goal of these two analyses is to examine the association of (i) living in a census tract with higher V-score with (ii) being virally nonsuppressed a year later, after adjusting for individual-level risk factors. Again, to keep things simple, we sample for each individual in the ALIVE study two visits about one year apart, where at the first visit the person was living in a Baltimore census tract for which we have a V-score, and at the second visit the person had a recorded viral load, resulting in a sample of 383 persons. The Analysis 2 column of Table 4 summarizes the baseline covariates relevant to the analysis.

**Table 4:** Summary of sample baseline characteristics in the analyses with V-score as exposure

	Analysis 2			Analysis 3			
	Full sample (n = 383)			Low V-score (n = 128)		High V-score (n = 133)	
	*mean*	*(SD)*		*mean*	*(SD)*	*mean*	*(SD)*
Age	52.6	(6.62)		53.4	(6.13)	51.5	(7.17)
Sex	*number*	*(percent)* ^*a*^		*number*	*(percent)* ^*a*^	*number*	*(percent)* ^*a*^
male	258	(67.4)		92	(71.9)	86	(64.7)
female	125	(32.6)		36	(28.1)	47	(35.3)
On antiretroviral treatment							
yes	295	(77.0)		103	(80.5)	92	(69.2)
no	84	(21.9)		23	(18.0)	40	(30.1)
Viral non-suppression							
yes	128	(33.4)		37	(28.9)	58	(43.6)
no	250	(65.3)		88	(66.8)	74	(55.6)
	*max*			*max*		*max*	
Viral load	550 K			525 K		550 K	
	*mean*	*(SD)*		*mean*	*(SD)*	*mean*	*(SD)*
CD4 count	417	(266)		427	(262)	403	(271)
Annual income < $5K	*number*	*(percent)* ^*a*^		*number*	*(percent)* ^*a*^	*number*	*(percent)* ^*a*^
yes	259	(67.6)		80	(62.5)	96	(72.2)
no	115	(30.0)		44	(34.4)	36	(27.1)
Health insurance coverage^b^							
yes	368	(96.1)		126	(98.4)	125	(94.0)
no	13	(3.4)		2	(1.6)	7	(5.3)
Elevated depressive symptoms^c^							
yes	89	(23.2)		24	(18.8)	32	(24.1)
no	292	(76.2)		103	(80.5)	100	(75.2)
Injection drug use^b^							
yes	104	(27.2)		29	(22.7)	44	(33.1)
no	276	(72.1)		98	(76.6)	87	(65.4)
Crack cocaine use^b^							
yes	83	(21.7)		23	(18.0)	32	(24.1)
no	298	(77.8)		104	(81.3)	100	(75.2)
Alcohol/drug treatment^b^							
yes	164	(42.8)		46	(35.9)	65	(48.9)
no	216	(56.4)		82	(64.1)	67	(50.4)
Any time in jail^b^							
yes	14	(3.7)		3	(2.3)	10	(7.5)
no	334	(87.2)		118	(92.2)	113	(85.0)
Any time homeless^b^							
yes	30	(7.8)		11	(8.6)	13	(9.8)
no	349	(91.1)		115	(89.8)	119	(89.5)
Number of moves^b^							
0	305	(79.6)		106	(82.8)	101	(75.9)
1	35	(9.1)		10	(7.8)	14	(10.5)
2	41	(10.7)		11	(8.6)	17	(12.8)

SD = standard deviation

^a^ Proportions may not add up to 100% due to missing data

^b^ These variables are about the past 6 months

^c^ Defined as scoring 23 + on the CES-D scale [45] for the past week

With the continuous exposure variable, a researcher might choose to use regression analysis. We performed this analysis by first imputing missing covariates, and then fitting a logit model and pooling coefficients across imputed datasets. This analysis estimates that after adjusting for individual-level covariates, a difference in V-score of one standard deviation is associated with an OR of 1.15 for viral non-suppression one year later, 95% CI = (0.86, 1.54), p-value = 0.33.

**Fig. 7:**
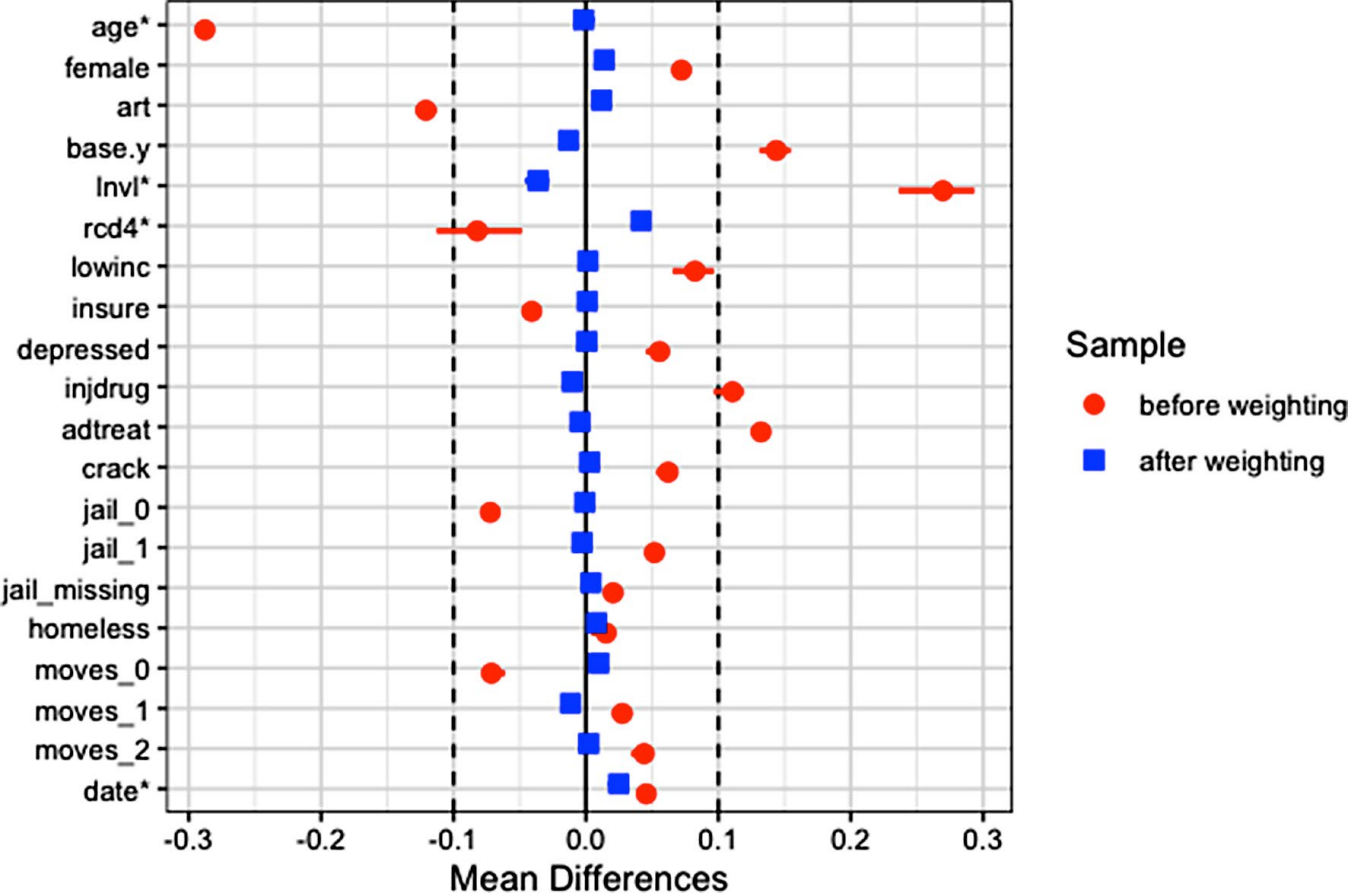
Analysis 3 – Covariate balance before and after weighting between high and low V-score groups. The horizontal bars show variation across imputed datasets. Mean differences for continuous variables (marked with *) are standardized

Another researcher may be hesitant to use regression adjustment due to concern about V-score and covariates being correlated, and instead would like to conduct an analysis where confounding is controlled via covariate balancing, like in Analysis 1. They may split the V-score variable into three equal-size bins and consider the difference between being in the top and bottom bins. The Analysis 3 part of Table 4 describes the high and low V-score subsamples. This analysis combines MI with propensity score weighting using the same method as in Analysis 1 but with high V-score as the exposure. Figure 7 shows that the weighting obtains covariate balance. On the OR scale, this analysis estimates that conditional on individual-level covariates, high (vs. low) V-score exposure is associated with an OR for viral non-suppression one year later of 1.16, 95% CI = (0.62, 2.16), p-value = 0.65. On the RD scale, high (vs. low) V-score exposure is associated with a RD for viral non-suppression one year later of 5.2 percentage points, 95% CI = (-7.7, 18.1), p-value = 0.43.

## Discussion

In this paper we proposed a characterization of place for health research where the multivariate measurement of place is distilled into a single score representing vulnerability to an outcome of interest (here viral non-suppression) based on place characteristics (here census tract-level measures of crime/policing events, and economic and housing conditions). We implemented this approach in a specific case of estimating V-score using HIV outcome data from a cohort of Black people with drug injection histories in Baltimore city, and illustrated the utility of the estimated V-score through some subsequent analyses.

A tangible output of this work is the set of estimated V-scores for Baltimore city census tracts, which could be of practical use for HIV researchers who wish to have a variable reflecting census tract level vulnerability to poor HIV outcomes. These scores are estimated for census tracts, which are larger and less heterogeneous than census block groups and other geo-units that may better reflect local social and economic dynamics. However, place data are more reliable and outcome data are less sparse at census tract level. Where data are richer and more abundant, V-score estimation for smaller spatial scales should be attempted.

We now offer a few general comments on V-score estimation methods and on the usefulness of V-scores in HIV research.

### On methods for V-score estimation

In this particular application, the methods we use for V-score estimation handle the complicated nature of the data by breaking it into multiple methodological issues and addressing them one by one. Using random forests as the modeling and prediction tool, we crafted a procedure tailored to the problem at hand. Because the data in each application is different (as evident in the diverse literature on place and HIV), the estimation procedure ultimately needs to respond to the features of the specific data. That said, this demonstration provides an example for how existing data, often complicated in one way or another, may be leveraged for the purpose of estimating place-based V-scores, and showcases how some issues which are quite common may be handled.

A detail should be made explicit for complete clarity: V-score estimation does not involve the spatial features of the data, specifically, the models for V-score estimation use as inputs the census tracts’ characteristics captured in the $X$ variables (crime/policing, housing and economic deprivation) but not the census tracts’ locations relative to one other. The reason is that (as mentioned earlier) the V-score is not an estimate of the outcome for a specific place, but is the expectation of the outcome for any place with such values of the $X$ variables. While spatial information might help explain more of outcome variation, it does not serve this purpose.

The methods in this paper produce a V-score that is specific to one outcome (here HIV viral non-suppression). There are times though when there may also be interest in dimension reduction of place characteristics that captures vulnerability to a range of outcomes (e.g., those along the HIV prevention-and-care continuum). Hence a natural extension to be investigated in future work is how to construct a V-score for multiple related outcomes. This could take either a two-step or a joint modeling approach. With the two-step approach one estimates for each outcome a V-score and then combine (e.g., using PCA on the V-scores). With the joint modeling approach, one could adopt a model where the place characteristics are causes of a latent variable which causes all the outcomes. This could be modeled parametrically via traditional structural equation modeling, or it could be modeled nonparametrically (which is the strategy of the current paper) using machine learning structures that involve latent variables such as neural networks.

### On utility of V-score in HIV research

The illustrative examples show two different uses of V-score, reflecting two different roles played by place. In the first example, the V-score is a covariate that is used to control place-level confounding of the association between an individual-level exposure (IDU) and the outcome (HIV viral non-suppression). We want to draw attention to the difference between adjusting and not adjusting for the V-score in the example: not adjusting for the V-score would inflate the exposure-outcome association. This is a classic example of effect estimate changing as we adjust for a confounder.

This simple example highlights a point that in a sense should be obvious but is often not considered (as mentioned in the Introduction section): when examining the effect of an individual-level exposure, place-level confounding should be considered. This matters because in many applications the exposure of interest is an individual behavior. The researcher may be looking at how much a negative behavior puts the person at risk for a poor outcome, or may be ultimately interested in how the outcome could be improved by changing a risk behavior. Not accounting for place-level confounding may lead to over-estimation of the effect of the individual’s behavior or an overly optimistic view of how much a behavior change may positively affect an outcome. The availability of a V-score for the outcome provides a simple way to adjust for that confounding by place factors and obtain more valid and realistic effect estimates.

It should be mentioned that another possibility for controlling place-level confounding is to use a V-score for the exposure (here injection drug use) if a V-score for the outcome is not available. The rationale is that such a V-score captures elements of place (contained in the place measurements) that are relevant to the exposure, so adjusting for the V-score adjusts for those elements. The “remaining” elements may be relevant to the outcome but since they are not relevant to the exposure are less likely to have a confounding effect. Which V-score type works better (i.e., is more effective in removing confounding), or when one works better than the other, is a question for future research.

A related role to be considered for place is as a moderator of individual-level exposure-outcome relationships. Such analyses could inform interventions targeting the individual-level exposures as well as interventions that both address individual-level exposures and intervene on structural factors that worsen or mitigate their effects. Also promising is the potential use of V-scores to examine place-based vulnerability as a mediator. In studies tracking residential relocation due to housing programs or natural disasters, initial poor health is associated with relocation to under-resourced communities [51–53]. This suggests that effects of poor health on long-term outcomes may be mediated by the experience of place-based vulnerability resulting from “health selection into neighborhoods” [52]. More broadly, settings where people are triggered to move present opportunities to study place-based vulnerability mediating causal effects.

Illustrative analyses 2 and 3 treat V-score as the exposure variable, examining whether living in higher vulnerability census tracts is associated with worse HIV outcome. This is closely related to analyses often seen in the place and HIV literature (and the majority of the work we have cited), which treat place characteristics as the risk factor of main interest. The only difference is how place is characterized. Putting the specific analyses aside and considering this strand of research generally, here the addition of the V-score to the HIV researcher’s toolbox provides another view of the effect of place. Even when we limit the notion of place to the collection of available variables, there is no one representation for the effect of that collection of variables on the outcome. With a measurement-centric characterization of place, we are looking at a slice of the complexity of place along specific pre-defined dimensions, e.g., summary measure of crime/policing activities (or of housing conditions, or of economic deprivation), or a composite of all three combined. With the current outcome-centric characterization of place, we have another slice of place in a possibly different dimension, one that is most relevant to the outcome.

It is important to note that for analyses where place is the exposure (or a moderator or mediator), whether to choose the measurement-centric or the outcome-oriented characterization of place should be determined by the research question. If the research question is about a specific dimension of place, e.g., how neighborhood economic deprivation puts people at risk for a given outcome, then the measurement-centric approach should be used where the exposure variable is a summary measure of economic deprivation (a D-score, not a V-score). If the interest is more generally what is the effect of place on the outcome, where place is captured by a collection of possibly multidimensional measures (and the dimensions themselves are *not* of interest), then we recommend the V-score approach, as the V-score is a summary measure that captures elements of place that matter to the outcome.

## Conclusion

To conclude, we proposed a useful characterization of place in the form of a vulnerability score, based on place measurements, for a specific outcome of interest. Using data from a cohort of people with histories of injecting drugs and administrative census tract data, we estimated the V-score for HIV viral non-suppression for Baltimore census tracts. This provides an example for how existing sources of data, which are often complicated, can be leveraged for the purpose of V-score estimation. We provide some illustrative analyses using V-score to represent place, and discuss the potential utility of V-scores in analyses of HIV outcomes, where place can play the role of exposure, confounder, moderator or even mediator.

### Electronic supplementary material

Below is the link to the electronic supplementary material.

**Supplementary Material 1:** Appendix A

**Supplementary Material 2:** Appendix B

## Data Availability

The data are not public and available upon request. Please contact Dr. Sabriya Linton (slinton1@jhu.edu).

## Code availability

Code is provided through the R-package placeVscore, available from https://github.com/trangnguyen74/placeVscore.

## Declarations

### Human accordance statement

The study was carried out in accordance with relevant guidelines and regulations, and in accordance with the Declaration of Helsinki.
