# Supplementary material for: Estimation of place-based vulnerability scores for HIV viral non-suppression: an application leveraging data from a cohort of people with histories of using drugs
Source: BMC Med Res Methodol. 2024 Jan 25;24:21. doi: 10.1186/s12874-023-02133-x (PMC10809603; doi:10.1186/s12874-023-02133-x)
Supplement: Supplementary file 1 — Supplementary Material 1: Appendix A [file 12874_2023_2133_MOESM1_ESM.html]

Appendix B: Illustration of V-score use


# Appendix B: Illustration of V-score use

### to article: Estimation of place-based vulnerability scores for an outcome: an application leveraging data on HIV viral non-suppression among people with histories of using drugs

```
library(tidyverse)
library(placeVscore)
library(cobalt)
```

# Analysis 1: V-score as exposure, logistic regression

```
vdat1 <- placeVscore:::ALIVE_illustrative.analysis.data(exposure = "vscore",
                                                        outcome  = "detect")

vdat1 <- vdat1 %>%
    mutate(vs      = vscore, 
           detect  = factor(detect),
           bdetect = factor(bdetect)) %>%
    select(-c(vscore, avscore))
```

## Descriptive statistics

```
tmp <- vdat1 %>%
    mutate(vl    = exp(lnvl),
           cd4   = rcd4^2) %>%
    select(-c(fid, ct, fdate, lnvl, rcd4,
              vs, detect)) %>%
    relocate(age, cd4, vl, 
             female, bdetect, lowinc, insure,
             depressed, injdrug, crack, adtreat, jail, homeless, moves)

table1::table1(~ ., data = tmp)
```

|  | Overall (N=383) |
| --- | --- |
| age |  |
| Mean (SD) | 52.6 (6.62) |
| Median [Min, Max] | 53.2 [37.0, 71.4] |
| cd4 |  |
| Mean (SD) | 417 (266) |
| Median [Min, Max] | 385 [0, 1500] |
| Missing | 7 (1.8%) |
| vl |  |
| Mean (SD) | 19000 (63300) |
| Median [Min, Max] | 40.0 [40.0, 550000] |
| Missing | 5 (1.3%) |
| female |  |
| 0 | 258 (67.4%) |
| 1 | 125 (32.6%) |
| bdetect |  |
| 0 | 200 (52.2%) |
| 1 | 178 (46.5%) |
| Missing | 5 (1.3%) |
| lowinc |  |
| 0 | 115 (30.0%) |
| 1 | 259 (67.6%) |
| Missing | 9 (2.3%) |
| insure |  |
| 0 | 13 (3.4%) |
| 1 | 368 (96.1%) |
| Missing | 2 (0.5%) |
| depressed |  |
| 0 | 292 (76.2%) |
| 1 | 89 (23.2%) |
| Missing | 2 (0.5%) |
| injdrug |  |
| 0 | 276 (72.1%) |
| 1 | 104 (27.2%) |
| Missing | 3 (0.8%) |
| crack |  |
| 0 | 298 (77.8%) |
| 1 | 83 (21.7%) |
| Missing | 2 (0.5%) |
| adtreat |  |
| 0 | 216 (56.4%) |
| 1 | 164 (42.8%) |
| Missing | 3 (0.8%) |
| jail |  |
| 0 | 334 (87.2%) |
| 1 | 14 (3.7%) |
| Missing | 35 (9.1%) |
| homeless |  |
| 0 | 349 (91.1%) |
| 1 | 30 (7.8%) |
| Missing | 4 (1.0%) |
| moves |  |
| 0 | 305 (79.6%) |
| 1 | 35 (9.1%) |
| 2 | 41 (10.7%) |
| Missing | 2 (0.5%) |
| byear |  |
| Mean (SD) | 2010 (2.38) |
| Median [Min, Max] | 2010 [2010, 2020] |

rm(tmp)

This `table1` function has the weird behavior of fixing
number of meaningful digits at 3, thus messing up the summary of year.
We need to get it the usual way.

```
summary(vdat1$byear)
#>    Min. 1st Qu.  Median    Mean 3rd Qu.    Max. 
#>    2009    2010    2012    2012    2014    2016
sd(vdat1$byear)
#> [1] 2.381149
```

## Multiple imputation

We need to deal with missing data. Let’s look at the pattern.

```
tmp <- mice::md.pattern(vdat1)
```

```
rm(tmp)
```

The most missingness is in variable jail. Looking at the variable
itself in the descriptive table, only a small percentage has jail=1.
This means multiple imputation may result in that variable being imputed
to 0 most of the time. Also, since this is a sensitive question, we are
concerned that missingness may be informative. Therefore, rather than
imputing this variable, we create a third category for missing jail.

```
vdat1 <- vdat1 %>% 
    mutate(jail = ifelse(is.na(jail), 3, jail),
           jail = factor(jail, labels = c("0", "1", "missing")))

tmp <- mice::md.pattern(vdat1)
```

```
rm(tmp)
```

```
vdat1 <- vdat1 %>%
    relocate("fid", "ct", "byear", 
             "age", "female", "jail", "fdate", "detect", "vs",
             # variables to be imputed in increasing missingness order
             "insure", "depressed", "crack", "moves", 
             "injdrug", "adtreat", "homeless",
             "bdetect", "lnvl",
             "rcd4", "lowinc")

imp.init <- mice::mice(vdat1, maxit = 0)

pred <- imp.init$predictorMatrix
meth <- imp.init$method

# exclude these variables from imputation models
pred[, c("fid", "ct", "byear")] <- 0

# trick to make sure lnvl is constant for bdetect==0
# (necessary b/c package mice does not allow imputation based on conditions)
pred["bdetect", "lnvl"] <- 0
meth["lnvl"] <- "cart"

rm(imp.init)
```

```
vdat1.imp <- mice::mice(vdat1, 
                        m = 50,
                        predictorMatrix = pred,
                        method          = meth,
                        print = F,
                        seed = nrow(vdat1))
```

The plots below show no problem with mixing and convergence are
good.

```
plot(vdat1.imp)
```

## Logistic regression

```
vdat1.fit <- mice::glm.mids(detect ~ bdetect + lnvl + rcd4 + age + female + lowinc +
                                insure + depressed + injdrug + adtreat + crack + 
                                jail + homeless + moves + fdate + vs, 
                            data = vdat1.imp, 
                            family = "binomial")
#> Warning: Use with(imp, glm(yourmodel).
vdat1.fit <- summary(mice::pool(vdat1.fit), conf.int = TRUE)
```

Estimated odds ratio for one percentage-point difference in
V-score

```
exp(vdat1.fit[, c("estimate", "2.5 %", "97.5 %")][vdat1.fit$term=="vs",])
#>    estimate     2.5 %   97.5 %
#> 19 1.022837 0.9792616 1.068351
```

# Analysis 2: high V-score as exposure, covariate balancing

```
vdat2 <- placeVscore:::ALIVE_illustrative.analysis.data(exposure = "vscore",
                                                        outcome  = "detect")

vdat2 <- vdat2 %>%
    mutate(vs      = avscore,
           detect  = factor(detect),
           bdetect = factor(bdetect)) %>%
    select(-c(vscore, avscore))

terts <- quantile(vdat2$vs, probs = c(1/3, 2/3))
terts
#> 33.33333% 66.66667% 
#>  1.409473  6.122993

vdat2 <- vdat2 %>%
    mutate(vs.hi = ifelse(vs <= terts[1], 0,
                          ifelse(vs >= terts[2], 1, NA)),
           vs.hi = factor(vs.hi)) %>%
    filter(!is.na(vs.hi)) %>%
    select(-vs)

vdat2 <- vdat2 %>% 
    mutate(jail = ifelse(is.na(jail), 3, jail),
           jail = factor(jail, labels = c("0", "1", "missing")))
```

## Descriptive statistics

```
tmp <- vdat2 %>%
    mutate(vl = exp(lnvl),
           cd4 = rcd4^2) %>%
    select(-c(fid, ct, fdate, lnvl, rcd4, detect)) %>%
    relocate(age, vl, cd4,
             female, bdetect, lowinc, insure,
             depressed, injdrug, crack, adtreat, jail, homeless, moves, vs.hi)

table1::table1(~ . | vs.hi, data = tmp, overall = FALSE)
```

|  | 0 (N=132) | 1 (N=129) |
| --- | --- | --- |
| age |  |  |
| Mean (SD) | 52.9 (5.98) | 51.7 (7.43) |
| Median [Min, Max] | 53.3 [37.2, 69.5] | 52.0 [37.0, 71.4] |
| vl |  |  |
| Mean (SD) | 17400 (61700) | 22100 (63700) |
| Median [Min, Max] | 40.0 [40.0, 467000] | 170 [40.0, 550000] |
| Missing | 4 (3.0%) | 1 (0.8%) |
| cd4 |  |  |
| Mean (SD) | 424 (257) | 397 (272) |
| Median [Min, Max] | 408 [5.00, 1250] | 378 [0, 1300] |
| Missing | 4 (3.0%) | 1 (0.8%) |
| female |  |  |
| 0 | 94 (71.2%) | 85 (65.9%) |
| 1 | 38 (28.8%) | 44 (34.1%) |
| bdetect |  |  |
| 0 | 77 (58.3%) | 60 (46.5%) |
| 1 | 51 (38.6%) | 68 (52.7%) |
| Missing | 4 (3.0%) | 1 (0.8%) |
| lowinc |  |  |
| 0 | 41 (31.1%) | 35 (27.1%) |
| 1 | 86 (65.2%) | 92 (71.3%) |
| Missing | 5 (3.8%) | 2 (1.6%) |
| insure |  |  |
| 0 | 2 (1.5%) | 6 (4.7%) |
| 1 | 130 (98.5%) | 122 (94.6%) |
| Missing | 0 (0%) | 1 (0.8%) |
| depressed |  |  |
| 0 | 108 (81.8%) | 95 (73.6%) |
| 1 | 23 (17.4%) | 33 (25.6%) |
| Missing | 1 (0.8%) | 1 (0.8%) |
| injdrug |  |  |
| 0 | 100 (75.8%) | 83 (64.3%) |
| 1 | 31 (23.5%) | 45 (34.9%) |
| Missing | 1 (0.8%) | 1 (0.8%) |
| crack |  |  |
| 0 | 103 (78.0%) | 96 (74.4%) |
| 1 | 28 (21.2%) | 32 (24.8%) |
| Missing | 1 (0.8%) | 1 (0.8%) |
| adtreat |  |  |
| 0 | 76 (57.6%) | 68 (52.7%) |
| 1 | 56 (42.4%) | 60 (46.5%) |
| Missing | 0 (0%) | 1 (0.8%) |
| jail |  |  |
| 0 | 118 (89.4%) | 112 (86.8%) |
| 1 | 3 (2.3%) | 8 (6.2%) |
| missing | 11 (8.3%) | 9 (7.0%) |
| homeless |  |  |
| 0 | 117 (88.6%) | 116 (89.9%) |
| 1 | 12 (9.1%) | 12 (9.3%) |
| Missing | 3 (2.3%) | 1 (0.8%) |
| moves |  |  |
| 0 | 106 (80.3%) | 99 (76.7%) |
| 1 | 10 (7.6%) | 13 (10.1%) |
| 2 | 15 (11.4%) | 16 (12.4%) |
| Missing | 1 (0.8%) | 1 (0.8%) |
| byear |  |  |
| Mean (SD) | 2010 (2.32) | 2010 (2.34) |
| Median [Min, Max] | 2010 [2010, 2020] | 2010 [2010, 2020] |

rm(tmp)
vdat2 %>%
group\_by(vs.hi) %>%
summarize(byear.mean = mean(byear),
byear.sd = sd(byear),
byear.median = median(byear),
byear.min = min(byear),
byear.max = max(byear),
.groups = "drop") %>%
ungroup()
#> # A tibble: 2 × 6
#> vs.hi byear.mean byear.sd byear.median byear.min byear.max
#>      
#> 1 0 2012. 2.32 2012 2009 2016
#> 2 1 2012. 2.34 2012 2009 2016

## Multiple imputation

```
tmp <- mice::md.pattern(vdat2)
```

```
rm(tmp)
```

```
vdat2 <- vdat2 %>%
    relocate("fid", "ct", "byear", 
             "age", "female", "jail", "fdate", "detect", "vs.hi",
             # variables to be imputed in increasing missingness order
             "insure", "depressed", "crack", "moves", 
             "injdrug", "adtreat", "homeless",
             "bdetect", "lnvl",
             "rcd4", "lowinc")

imp.init <- mice::mice(vdat2, maxit = 0)

pred <- imp.init$predictorMatrix
meth <- imp.init$method

# exclude these variables from imputation models
pred[, c("fid", "ct", "byear")] <- 0

# trick to make sure lnvl is constant for bdetect==0
# (necessary b/c package mice does not allow imputation based on conditions)
pred["bdetect", "lnvl"] <- 0
meth["lnvl"] <- "cart"

rm(imp.init)
```

```
vdat2.imp <- mice::mice(vdat2, 
                        m = 50,
                        predictorMatrix = pred,
                        method          = meth,
                        print = F,
                        seed = nrow(vdat2))
```

```
plot(vdat2.imp)
```

## Propensity score analysis

```
vdat2.weighted <- MatchThem::weightthem(vs.hi ~ bdetect + lnvl + rcd4 + age + female + lowinc +
                                            insure + depressed + injdrug + adtreat + crack + 
                                            jail + homeless + moves + fdate, 
                                        datasets = vdat2.imp, estimand = "ATE",
                                        approach = "within", method = "ps")
#> Estimating weights     | dataset: #1 #2 #3 #4 #5 #6 #7 #8 #9 #10 #11 #12 #13 #14 #15 #16 #17 #18 #19 #20 #21 #22 #23 #24 #25 #26 #27 #28 #29 #30 #31 #32 #33 #34 #35 #36 #37 #38 #39 #40 #41 #42 #43 #44 #45 #46 #47 #48 #49 #50
```

```
cobalt::bal.tab(vdat2.weighted)
#> Balance summary across all imputations
#>                  Type Min.Diff.Adj Mean.Diff.Adj Max.Diff.Adj
#> prop.score   Distance      -0.0472       -0.0328      -0.0215
#> bdetect        Binary      -0.0155       -0.0124      -0.0097
#> lnvl          Contin.      -0.0503       -0.0412      -0.0327
#> rcd4          Contin.       0.0249        0.0305       0.0341
#> age           Contin.       0.0053        0.0088       0.0126
#> female         Binary       0.0114        0.0138       0.0161
#> lowinc         Binary       0.0020        0.0042       0.0063
#> insure         Binary       0.0016        0.0037       0.0067
#> depressed      Binary      -0.0068       -0.0033       0.0006
#> injdrug        Binary      -0.0126       -0.0094      -0.0058
#> adtreat        Binary      -0.0029       -0.0006       0.0016
#> crack          Binary      -0.0069       -0.0033      -0.0003
#> jail_0         Binary       0.0017        0.0045       0.0069
#> jail_1         Binary      -0.0098       -0.0074      -0.0045
#> jail_missing   Binary       0.0010        0.0029       0.0045
#> homeless       Binary      -0.0005        0.0009       0.0041
#> moves_0        Binary       0.0041        0.0074       0.0102
#> moves_1        Binary      -0.0135       -0.0116      -0.0090
#> moves_2        Binary       0.0024        0.0043       0.0069
#> fdate         Contin.       0.0070        0.0120       0.0163
#> 
#> Average effective sample sizes across imputations
#>            Control Treated
#> Unadjusted  132.    129.  
#> Adjusted    115.87  121.71
cobalt::love.plot(vdat2.weighted, thresholds = .1, 
                  stars = "std")
```

```
vdat2.wtd.fit <- with(data = vdat2.weighted,
                      expr = glm(detect ~ bdetect + lnvl + rcd4 + age + female + lowinc + 
                                     insure + depressed + injdrug + adtreat + crack + jail +
                                     homeless + moves + fdate + vs.hi, 
                                 family = "quasibinomial")) 

vdat2.wtd.fit <- summary(mice::pool(vdat2.wtd.fit), conf.int = TRUE)

exp(vdat2.wtd.fit[, c("estimate", "2.5 %", "97.5 %")][vdat2.wtd.fit$term=="vs.hi1",])
#>    estimate     2.5 %   97.5 %
#> 19 1.479482 0.8034182 2.724441
```

# Analysis 3: current drug injection as exposure, V-score as covariate to be adjusted

```
idat <- placeVscore:::ALIVE_illustrative.analysis.data(exposure = "injdrug", 
                                                       outcome  = "detect")

idat <- idat %>%
    mutate(vs      = avscore,
           detect  = factor(detect),
           bdetect = factor(bdetect)) %>%
    select(-c(vscore, avscore)) %>% 
    
    mutate(jail = ifelse(is.na(jail), 3, jail),
           jail = factor(jail, labels = c("0", "1", "missing"))) %>%
    select(-c(crack, adtreat))
```

## Descriptive statistics

```
tmp <- idat %>%
    mutate(vl = exp(lnvl),
           cd4 = rcd4^2) %>%
    select(-c(fid, ct, fdate, lnvl, rcd4, detect)) %>%
    relocate(age, vl, cd4,
             female, bdetect, lowinc, insure,
             depressed, injdrug, jail, homeless, moves, vs)

table1::table1(~ . | injdrug, data = tmp, overall = FALSE)
```

|  | 0 (N=295) | 1 (N=110) |
| --- | --- | --- |
| age |  |  |
| Mean (SD) | 53.0 (6.56) | 51.3 (6.52) |
| Median [Min, Max] | 53.1 [38.2, 69.4] | 51.9 [27.5, 65.6] |
| vl |  |  |
| Mean (SD) | 13200 (65400) | 21600 (73700) |
| Median [Min, Max] | 40.0 [40.0, 930000] | 192 [40.0, 702000] |
| Missing | 3 (1.0%) | 2 (1.8%) |
| cd4 |  |  |
| Mean (SD) | 450 (296) | 344 (242) |
| Median [Min, Max] | 378 [0, 1750] | 283 [10.0, 1130] |
| Missing | 5 (1.7%) | 0 (0%) |
| female |  |  |
| 0 | 198 (67.1%) | 78 (70.9%) |
| 1 | 97 (32.9%) | 32 (29.1%) |
| bdetect |  |  |
| 0 | 180 (61.0%) | 43 (39.1%) |
| 1 | 112 (38.0%) | 65 (59.1%) |
| Missing | 3 (1.0%) | 2 (1.8%) |
| lowinc |  |  |
| 0 | 93 (31.5%) | 28 (25.5%) |
| 1 | 198 (67.1%) | 80 (72.7%) |
| Missing | 4 (1.4%) | 2 (1.8%) |
| insure |  |  |
| 0 | 9 (3.1%) | 6 (5.5%) |
| 1 | 286 (96.9%) | 103 (93.6%) |
| Missing | 0 (0%) | 1 (0.9%) |
| depressed |  |  |
| 0 | 241 (81.7%) | 78 (70.9%) |
| 1 | 54 (18.3%) | 32 (29.1%) |
| jail |  |  |
| 0 | 261 (88.5%) | 92 (83.6%) |
| 1 | 10 (3.4%) | 11 (10.0%) |
| missing | 24 (8.1%) | 7 (6.4%) |
| homeless |  |  |
| 0 | 276 (93.6%) | 94 (85.5%) |
| 1 | 18 (6.1%) | 15 (13.6%) |
| Missing | 1 (0.3%) | 1 (0.9%) |
| moves |  |  |
| 0 | 240 (81.4%) | 80 (72.7%) |
| 1 | 28 (9.5%) | 14 (12.7%) |
| 2 | 27 (9.2%) | 16 (14.5%) |
| vs |  |  |
| Mean (SD) | 2.78 (4.69) | 3.87 (4.74) |
| Median [Min, Max] | 2.99 [-7.62, 11.4] | 4.58 [-7.31, 11.4] |
| Missing | 33 (11.2%) | 5 (4.5%) |
| byear |  |  |
| Mean (SD) | 2010 (2.34) | 2010 (2.51) |
| Median [Min, Max] | 2010 [2010, 2020] | 2010 [2010, 2020] |

rm(tmp)
idat %>%
group\_by(injdrug) %>%
summarize(byear.mean = mean(byear),
byear.sd = sd(byear),
byear.median = median(byear),
byear.min = min(byear),
byear.max = max(byear),
.groups = "drop") %>%
ungroup()
#> # A tibble: 2 × 6
#> injdrug byear.mean byear.sd byear.median byear.min byear.max
#>      
#> 1 0 2012. 2.34 2012 2009 2016
#> 2 1 2012. 2.51 2012 2009 2016

## Multiple imputation

```
tmp <- mice::md.pattern(idat)
```

```
rm(tmp)
```

```
idat <- idat %>%
    relocate("fid", "ct", "byear", 
             "injdrug", "age", "female", "jail", "fdate", "detect", "depressed", "moves",
             # variables to be imputed in increasing missingness order
             "insure", "homeless", 
             "bdetect", "lnvl", "rcd4",
             "lowinc", "vs")

imp.init <- mice::mice(idat, maxit = 0)

pred <- imp.init$predictorMatrix
meth <- imp.init$method

# exclude these variables from imputation models
pred[, c("fid", "ct", "byear")] <- 0
pred["ct", ] <- 0
meth["ct"] <- ""

# trick to make sure lnvl is constant for bdetect==0
# (necessary b/c package mice does not allow imputation based on conditions)
pred["bdetect", "lnvl"] <- 0
meth["lnvl"] <- "cart"

rm(imp.init)
```

```
idat.imp <- mice::mice(idat, 
                       m = 50,
                       predictorMatrix = pred,
                       method          = meth,
                       print = F,
                       seed = nrow(idat))
```

```
plot(idat.imp)
```

## Propensity score analysis

```
idat.weighted <- MatchThem::weightthem(injdrug ~ bdetect + lnvl + rcd4 + age + female + lowinc +
                                           insure + depressed + 
                                           jail + homeless + moves + fdate + vs, 
                                       datasets = idat.imp, estimand = "ATE",
                                       approach = "within", method = "gbm")
#> Estimating weights     | dataset: #1 #2 #3 #4 #5 #6 #7 #8 #9 #10 #11 #12 #13 #14 #15 #16 #17 #18 #19 #20 #21 #22 #23 #24 #25 #26 #27 #28 #29 #30 #31 #32 #33 #34 #35 #36 #37 #38 #39 #40 #41 #42 #43 #44 #45 #46 #47 #48 #49 #50
```

```
cobalt::bal.tab(idat.weighted)
#> Balance summary across all imputations
#>                  Type Min.Diff.Adj Mean.Diff.Adj Max.Diff.Adj
#> prop.score   Distance       0.8802        1.0861       1.2925
#> bdetect        Binary       0.0477        0.0614       0.0745
#> lnvl          Contin.       0.0662        0.0799       0.1083
#> rcd4          Contin.      -0.1412       -0.1136      -0.0933
#> age           Contin.      -0.1319       -0.1048      -0.0804
#> female         Binary      -0.0770       -0.0632      -0.0511
#> lowinc         Binary       0.0043        0.0208       0.0412
#> insure         Binary      -0.0284       -0.0167      -0.0128
#> depressed      Binary       0.0444        0.0508       0.0577
#> jail_0         Binary      -0.0268       -0.0214      -0.0161
#> jail_1         Binary       0.0235        0.0254       0.0291
#> jail_missing   Binary      -0.0082       -0.0040       0.0000
#> homeless       Binary       0.0311        0.0349       0.0396
#> moves_0        Binary      -0.0388       -0.0269      -0.0120
#> moves_1        Binary       0.0007        0.0102       0.0222
#> moves_2        Binary       0.0060        0.0167       0.0260
#> fdate         Contin.       0.0233        0.0394       0.0546
#> vs            Contin.       0.0154        0.0530       0.0912
#> 
#> Average effective sample sizes across imputations
#>            Control Treated
#> Unadjusted  295.    110.  
#> Adjusted    288.04   86.62
cobalt::love.plot(idat.weighted, thresholds = .1, 
                  stars = "std")
```

```
idat.wtd.fit <- with(data = idat.weighted,
                     expr = glm(detect ~ bdetect + lnvl + rcd4 + age + female + lowinc + 
                                    insure + depressed + jail +
                                    homeless + moves + fdate + vs + injdrug, 
                                family = "quasibinomial")) 

idat.wtd.fit <- summary(mice::pool(idat.wtd.fit), conf.int = TRUE)

exp(idat.wtd.fit[, c("estimate", "2.5 %", "97.5 %")][idat.wtd.fit$term=="injdrug1",])
#>    estimate     2.5 %   97.5 %
#> 17  1.34143 0.7941322 2.265913
```
