# Supplementary material for: Estimation of place-based vulnerability scores for HIV viral non-suppression: an application leveraging data from a cohort of people with histories of using drugs
Source: BMC Med Res Methodol. 2024 Jan 25;24:21. doi: 10.1186/s12874-023-02133-x (PMC10809603; doi:10.1186/s12874-023-02133-x)
Supplement: Supplementary file 2 — Supplementary Material 2: Appendix B [file 12874_2023_2133_MOESM2_ESM.html]

Appendix A: V-score estimation


# Appendix A: V-score estimation

### to article: Estimation of place-based vulnerability scores for an outcome: an application leveraging data on HIV viral non-suppression among people with histories of using drugs

- readme
- Introduction
- Data
  - Place data
  - Outcome and target
    population
  - Outcome data
- V-score
  estimation
  - A brief introduction to
    random forests
  - Method
    issue 1: The place characteristics data is clustered in place
    units
  - Method issue
    2: Places are connected by individuals
  - Method
    issue 3: Outcome data is clustered in individuals and
    unbalanced
  - Method
    issue 4: Some place variables are not available for all years
  - Method issue 5:
    Time trends in the outcome data
  - Consideration 6: average
    Vscore

# readme

More details of the work is shown here than in the article. To make
this content simple to follow as you read the article, this document
will track the structure of the article. This appendix (Appendix A)
covers through the estimation of the V-scores. Appendix B covers the
illustrative analyses using the V-scores.

The code used to make figures that appear in the article are included
here for transparency, but those specific figures are not included. Only
figures that don’t appear in the paper are shown here.

This document uses `R` version 4.1.2,
`tidyverse` version 1.3.1, `grf` version 2.0.2,
and `placeVscore` version 0.1.0.

```
library(tidyverse)
library(grf)
library(placeVscore)
```

```
figures4paper.dir <- "~/OneDrive - Johns Hopkins/placeVscore/manuscript/figures"
```

# Introduction

No computing here.

# Data

Let us load the data for our example into two objects
`pdat` (place data) and `ydat` (outcome data).

The data are not publicly avaiilable so we will show the data
structure, summary statistics, and analysis results, but will be careful
not show data values. (The two functions below only work on a computer
that has the raw data files.)

```
pdat <- placeVscore:::Baltimore_place.data_long(place = "ct")
ydat <- placeVscore:::ALIVE_hiv.nonsuppression.data(place = "ct")
```

We describe the datasets below so this code can be adapted to do
similar analyses on other data.

## Place data

#### Variables and sources

```
length(unique(pdat$place))
#> [1] 200
sort(unique(pdat$time))
#>  [1] 2000 2001 2002 2003 2004 2005 2006 2007 2008 2009 2010 2011 2012 2013 2014
#> [16] 2015 2016 2017
```

`pdat` includes two variables `place` and
`time`, which together define data units. This is a common
structure of place data, where the place characteristics are measured
repeatedly over time. In the current example, `place` means
census tract (there are 200 census tracts), and `time` means
year (ranging from 2000 to 2017). We keep the label generic, so that the
code can be used for other definitions of place and time.

```
names(pdat)
#>  [1] "place"        "time"         "part1"        "violent"      "gunhom"      
#>  [6] "narcotic"     "shoot"        "domvio"       "juvedrug"     "foreclose"   
#> [11] "ownoccupy"    "renovate"     "vacant"       "codeviol"     "lowwage"     
#> [16] "poverty"      "femalehh"     "pubassist"    "nohighschool" "unemploy"    
#> [21] "nocar"
```

The other variables in `pdat` include crime event counts
(`part1` through `juvedrug`), measures of housing
conditions (`foreclose` through `codeviol`), and
measures of economic deprivation (`lowwage` through
`nocar`) – see description in the article.

```
crime.vars <- c("part1", "violent", "gunhom", "narcotic", "shoot", "domvio", "juvedrug")
house.vars <- c("foreclose", "ownoccupy", "renovate", "vacant", "codeviol")
ecndp.vars <- c("lowwage", "poverty", "femalehh", "pubassist", "nohighschool", "unemploy", "nocar")
place.vars <- c(crime.vars, house.vars, ecndp.vars)
```

#### Availability of variables

It is important to note that not all place features are available for
all years, as shown below.

```
vars_available(data = pdat, vars = place.vars, by = "time"
               )$avail.plot +
    geom_rect(xmin = 0.5, xmax = 19.5, ymin = 9.5, ymax = 17.5, 
              fill = NA, color = "gray50", size = 1.5) +
    ggtitle("Availability of place variables")
#> Warning: Using `size` aesthetic for lines was deprecated in ggplot2 3.4.0.
#> ℹ Please use `linewidth` instead.
#> This warning is displayed once every 8 hours.
#> Call `lifecycle::last_lifecycle_warnings()` to see where this warning was
#> generated.
```

Due to a large number of variables not being available for 2001-2008
and for 2017, in this analysis we restrict to using data from 2009 to
2016 (the area in the box drawn on the plot above.

```
pdat <- subset(pdat, time %in% 2009:2016)
```

#### Distributions of variables

```
pdat %>%
    gather(key = "key", value = "value", -c(place, time)) %>%
    mutate(key = factor(key, levels = place.vars)) %>%
    
    ggplot(aes(x = time, y = value, group = time)) +
    geom_violin() +
    facet_wrap(~ key, scales = "free_y", ncol = 5) +
    labs(title = "Figure A: Density plots of place variables")
```

```
tmp <- pdat[place.vars]
box.index <- c(1,
               1 + length(crime.vars),
               1 + length(c(crime.vars, house.vars)),
               length(place.vars))

corrplot::corrplot(cor(tmp, use = "pairwise.complete.obs"), 
                   method = 'color',
                   tl.cex = .7,
                   cl.cex = .7,
                   title = "Correlation matrix of place variables",
                   mar = c(0,0,1,0)) %>%
    corrplot::corrRect(index = box.index)
```

If we do some work finding the right transformations for these
variables, we can find a stronger correlation matrix, for example, the
one using the code below. But for the current V-score purpose, we do not
need to concern ourselves with this.

```
tmp[crime.vars] <- log(tmp[crime.vars] + 1)
tmp[c("ownoccupy", "renovate")] <- -tmp[c("ownoccupy", "renovate")]
data.table::setnames(tmp,
                     old = crime.vars,
                     new = paste0("ln(", crime.vars, "+1)"))
data.table::setnames(tmp, 
                     old = c("ownoccupy", "renovate"),
                     new = paste0("rv.", c("ownoccupy", "renovate")))

corrplot::corrplot(cor(tmp, use = "pairwise.complete.obs"), 
                   method = 'color',
                   tl.cex = .7,
                   cl.cex = .7,
                   title = "Correlation matrix of transformed place variables",
                   mar = c(0,0,1,0)) %>%
    corrplot::corrRect(index = box.index)

rm(tmp, box.index, crime.vars, house.vars, ecndp.vars)
```

## Outcome and target population

No computing here.

## Outcome data

The outcome of interest is HIV viral nonsuppression, specifically
among Black people with a drug use history. For motivation of interest
in this population, see the article.

It is important to clarify what is the ideal data for the current
purpose, so that we are aware of how the available data compares to the
ideal. The ideal data for estimation of place-based vulnerability scores
for an outcome is prevalence of the outcome at the level of the place
data, i.e., for each census tract in each year; such data are not
available. The second ideal data is data appropriate for estimation of
such prevalence, i.e., a survey with samples representative of the
target population in each census tract in each year; such data are also
not available. As our current purpose is not to estimate prevalence,
there is a third ideal which is data representative of the target
population in Baltimore, even if the sample size may be not enough to
estimate prevalence for each and every census tract in each year.

What we have is data from the ALIVE study, an existing cohort that
was assembled not for the purpose of the current analysis. The hope is
that data from individuals who resided in a large number of census
tracts will provide some signal about place-level vulnerability based on
place characteristics. This data source has a longitudinal feature that
we will partially address in the analysis.

```
names(ydat)
#> [1] "place" "time"  "y"     "fid"
```

This data file `ydat` has been processed so it contains
only the variables we need. The first two variables are
`place` and `time`, which we will use to join with
`pdat`. Third is the outcome variable `y`, here a
binary variable indicating viral nonsuppression. (If the data were from
repeated cross-sectional surveys, these three variables would be
sufficient.) As this is a cohort, we include a fourth variable
`fid`, a fake ID variable that replaces the real ID. This
variable will be used to handle the fact that participants had repeated
outcome values.

```
ydat <- subset(ydat, time  %in% unique(pdat$time))
ydat <- subset(ydat, place %in% unique(pdat$place))

length(unique(ydat$place))
#> [1] 161
sort(unique(ydat$time))
#> [1] 2009 2010 2011 2012 2013 2014 2015 2016
```

We subset this data to Baltimore city census tracts (contained in the
place data), resulting in coverage of 161 of Baltimore’s 200 census
tracts. We also subset this data to the time span in
`pdat`.

A few numbers for the paper:

```
cat("Number of persons: ", length(unique(ydat$fid)), "\n")
#> Number of persons:  464
cat("Number of observations: ", nrow(ydat), "\n")
#> Number of observations:  3890
cat("Number of place-time cells: ", 
    ydat %>% select(place, time) %>% distinct() %>% nrow())
#> Number of place-time cells:  957
```

We can see in this raw data that there is a trend of declining
prevalence of viral nonsuppression.

```
ydat %>% 
    group_by(time) %>%
    summarize(y = mean(y)) %>%
    ungroup()
#> # A tibble: 8 × 2
#>    time     y
#>   <dbl> <dbl>
#> 1  2009 0.476
#> 2  2010 0.386
#> 3  2011 0.330
#> 4  2012 0.290
#> 5  2013 0.255
#> 6  2014 0.218
#> 7  2015 0.211
#> 8  2016 0.235
```

# V-score estimation

With place characteristics data connected to outcome data, we want to
fit a flexible model for the outcome using place characteristics and
time as predictors, and then use the model to predict for each place at
each time. The predicted values are based on place characteristics and
time, so we will need to do some processing to remove the time trend in
the predicted values before using them as V-scores, but the key idea is
to use the model-based predictions.

This `model.form`[ula] here contains `y` (the
outcome) plus time and place variables as predictors.

```
model.form <- paste("y ~ time +", paste(place.vars, collapse = " + "))
```

There are subtleties about the fitting of the model and the
computation of predicted values that we need to consider. Some of these
apply generally, others are specific to our current outcome data.

## A brief introduction to random forests

No computing here.

## Method issue 1: The place characteristics data is clustered in place units

When place characteristics data spans multiple time points (here
years), which is a common situation, such data is clustered in place
units. We thus wish to use a modeling tool that handles clustering.
While there are many software options for implementing random forests,
we choose the R package `grf` for it accommodates clustering
both (a) when drawing subsamples to grow trees (clusters are sampled
first and then the units within clusters) and (b) when computing
predictions (OOB prediction uses trees that do not include the clusters
being predicted for). (a) ensures that the resulting model is not too
confident due to wrongly treating the observations within clusters as
independent (thus mistaking that there is more information than there
actually is). (b) ensures that outcome data from a cluster is not
involved in predicting for the cluster.

If the outcome data comes from repeated surveys (not a cohort),
clustering in place units is the only issue, and one could fit a single
random forest (tuning parameters to minimize prediction error on unseen
data), use OOB prediction for place-time units present in the sample and
regular (aka out-of-sample, OOS) prediction for place-time units outside
the sample. The code for that procedure follows.

```
py.basic <- inner_join(pdat, ydat, by = c("place", "time"))

basic <- list()

py <- py.basic %>% collect_binary.y()

basic$rf <- 
    grf..regression_forest(formula     = model.form,
                           data        = py.basic,
                           cluster.var = "place",
                           
                           ci.group.size = 1,
                           sample.fraction = .7,
                           seed = 123)

basic$vi <- grf..variable_importance(basic$rf)

basic$ypreds <- get_ypred.oob(dat = py.basic, rf = basic$rf)
basic$ypreds <- get_ypred.oos(dat = pdat, rf = basic$rf, 
                               ypreds = basic$ypreds)

basic$rmse <- compute_rmse(ydat = ydat, ypreds = basic$ypreds)

rm(py.basic, py)
```

Here function `collect_binary.y()` collects outcome data
in each place-time cell into at most two rows, one for `y=1`
and one for `y=0`, and assign the corresponding counts to
variable `s.wt`.

Functions `grf..regression_forest()` and
`grf..variable_importance()` are our wrappers for the
`grf` package functions `grf::regression_forest()`
and `grf::variable_importance()` to allow using model formula
(instead of X and Y matrices) syntax and to label the variable
importance (VI) output with variable names.

Function `get_ypred.oob()` obtains OOB predictions for
in-sample place-time units. Function `get_ypred.oos()`
obtains OOS predictions for place-time units in `pdat` that
don’t appear in `ypreds` (here the data frame that holds
already computed OOB predictions).

The arguments `ci.group.size = 1` and
`sample.fraction = .7` indicate some of the parameters used
in the fitting the random forest. We discuss these briefly in the
paper.

## Method issue 2: Places are connected by individuals

In our illustrative example, the outcome data is not from repeated
surveys but from a cohort of individuals, who may be seen in multiple
places at different time points.

The key point of OOB prediction is that the prediction on a unit is
based only on the values of its regressors and does not touch its
outcome variable. The OOB prediction from the clustered random forest
above ensures that the prediction for census tract A is not based on
`y` values seen in census tract A. In our current example,
however, some individual may have lived in both census tracts A and B,
so their `y` values in the two census tracts are connected.
Therefore we want to exclude from the building of the model used to
predict for census tract A not only all `y` values seen in
census tract A, but also all `y` values of such a
census-tract-crossing individual that are seen elsewhere.

To achieve this, instead of simply using OOB prediction from a single
random forest as above, we manually implement a leave-one-out (LOO)
procedure where for one census tract at a time, we remove data of that
census tract and data from all persons connected to that census tract,
fit a random forest to the remaining data and use it to predict for the
left out census tract. This data processing uses function
`rm_place_and_connected_persons()`. The code for this method
is slightly more complicated and takes longer to run, as it requires
fitting one LOO random forest for each of the 161 census tracts that are
in the sample.

For the 39 out-of-sample census tracts, we would compute outcome
predictions based on a random forest fit to the whole sample as
above.

The code looks like below.

```
py.basic <- inner_join(pdat, ydat, by = c("place", "time"))

# census tracts in sample
places.in.sample <- unique(py.basic$place)

set.seed(456)
z.seeds <- sample(.Machine$integer.max, size = length(places.in.sample))

loo <- list()

loo$ypreds <- NULL
loo$vi <- NULL

for (z in 1:length(places.in.sample)) {
    
    # which place to predict on
    z.place <- places.in.sample[z]
    
    # the place's characteristics based on which to predict
    z.pdat <- subset(pdat, place==z.place) 
    
    # LOO data for model fitting
    z.py <- py.basic %>%  
        rm_place_and_connected_persons(place = z.place) %>%
        collect_binary.y()
    
    z.seed <- z.seeds[z]
    
    # LOO forest
    z.rf <- 
        grf..regression_forest(formula     = model.form,
                               data        = z.py,
                               cluster.var = "place",
                               
                               ci.group.size = 1,
                               sample.fraction = .7,
                               compute.oob.predictions = FALSE,
                               seed = z.seed)
    
    loo$vi <- cbind(loo$vi, grf..variable_importance(z.rf))
    colnames(loo$vi)[z] <- as.character(z.place)
    
    # compute ypreds for target census tract
    loo$ypreds <- get_ypred.oos(dat    = z.pdat,
                                rf     = z.rf,
                                ypreds = loo$ypreds,
                                loo    = TRUE)
    
    rm(z.place, z.pdat, z.py, z.seed, z.rf)
}

rm(z.seeds)

# compute ypreds for out-of-sample census tracts
loo$ypreds <- get_ypred.oos(dat    = pdat,
                            rf     = basic$rf,
                            ypreds = loo$ypreds)

# compute RMSE using the full py dataset earlier (not quite fair)
loo$rmse <- compute_rmse(ydat = ydat, loo$ypreds)
```

## Method issue 3: Outcome data is clustered in individuals and unbalanced

This feature of the data causes two problems: individuals who were
seen for many visits overall have more influence than those with few
visits; and individuals who stayed in the same census tract (or who move
around but within census tracts that have similar characteristics) have
high influence on that (those) census tract(s) and may skew the
results.

As a first step to reduce the problem of varying influence, within
each place-time cell, we downweights individuals that appear more than
once (coding a new variable `s.wt`) so each person has a
count of at most 1 in each cell. This is done using function
`norm_individual_cell.weights()`.

```
p1 <- ydat %>%
    group_by(fid) %>%
    summarize(obs = n()) %>%
    ungroup() %>%
    ggplot(aes(x = obs)) +
    geom_histogram(breaks = seq(.75, 17.25, .5)) +
    labs(x = "observation count", y = "number of persons")
p2 <- ydat %>%
    norm_individual_cell.weights() %>%
    group_by(fid) %>%
    summarize(num.place.times = sum(s.wt)) %>%
    ungroup() %>%
    ggplot(aes(x = num.place.times)) +
    geom_histogram(breaks = seq(.75, 17.25, .5)) +
    labs(x = "place-time count", y = "number of persons")

gridExtra::grid.arrange(p1, p2, ncol = 1);  rm(p1, p2)
```

Figure 1 in paper

```
fig1 <- ydat %>%
    group_by(fid) %>%
    summarize(obs = n()) %>%
    ungroup() %>%
    ggplot(aes(x = obs)) +
    geom_histogram(breaks = seq(.75, 17.25, .5)) +
    labs(x = "observation count", y = "number of persons") +
    theme_bw()
ggsave("numobs_histogram.png",
       plot = fig1,
       device = "png",
       path = figures4paper.dir,
       width = 6, height = 2,
       units = "in",
       dpi = 600)
```

This pares down some of the large individual weights, as shown in the
difference between the two histograms above, one for the individual’s
number of observations, the other for the individual’s number of
place-time cells.

It remains, however, that some individuals are present in the sample
for all time points, and for up to 14 place-time cells (if they move a
lot), while others are present for only one or two points.

The cleanest way to deal with this problem is to sample one data
point for each individual and discard all the rest. This completely
breaks the dependence of outcome data within individuals. However, it
drastically reduces sample size and throws away a lot of data, which is
not ideal.

We make a less extreme choice: to sample a maximum of three time
points for each individual, and sample one place per time point (if the
individual was seen in more than one place). This allows each individual
to contribute a maximum of three data points. Our motivation in making
this choice is to make the data more balanced and reduce the
disproportionate influence of long-term participants. We accept the
remaining dependence in the data to prevent too large a sample loss (the
resulting sample is a third of the naive sample size). This number 3 is
arbitrary though, and another number may be argued for.

As this is random sampling, to stabilize estimates, we want to do
this multiple times and average the predictions. We implement ten
repetitions. Each repetition includes one LOO random forest for
prediction on each of the in-sample 161 census tracts, and one non-LOO
random forest for prediction on the out-of-sample census tracts. The
code is written as a for loop over `s` which indexes the
repetition. Since the repetitions are separate, they can be parallelized
to speed up computing. Or they can be run one at a time and results from
each run can be saved to clear memory.

The average sample of these repetitions is represented in the top
panel of the plot below, and the raw sample in the bottom panel. Note
that the scale of place-time cell sample size differs between the two
plots. Roughly speaking, the range of cell size goes from one with much
variation (0 to 30) to one with less variation (0 to 8), with totals for
the years being 163.6, 165.5, 161, 158.3, 132.4, 119.9, 118.2, 148.1 for
years 2009 to 2016, respectively.

```
p1 <- plot_y.sample(ydat, balance = TRUE)
p2 <- plot_y.sample(ydat, balance = FALSE)
gridExtra::grid.arrange(p1, p2, ncol = 1);  rm(p1, p2)
```

Figure 2 in paper:

```
fig2 <- function(ydat,
                 sum.times = 3,
                 sum.places = 1) {

    place <- time <- y <- s.wt <- size <- NULL

    ydat.bal <- ydat %>% 
        balance_individual_data(sum.times = sum.times,
                                sum.places = sum.places) %>%
        mutate(version = "effective")
    
    ydat.raw <- ydat %>% mutate(s.wt = 1) %>%
        mutate(version = "raw")

    bind_rows(ydat.bal, ydat.raw) %>% 
        group_by(version, place, time) %>%
        mutate(version = factor(version, 
                                levels = c("raw", "effective"),
                                labels = c("RAW SAMPLE SIZE",
                                           "\nEXPECTED SAMPLE SIZE with rough balancing"))) %>%
        summarize(size = sum(s.wt),
                  .groups = "drop") %>%
        ungroup() %>%

        ggplot(aes(x = as.integer(as.factor(place)), y = time)) +
        geom_point(aes(size = size, color = size), alpha = .4) +
        theme_minimal() +
        theme(legend.position = "right",
              legend.box.just = "center",
              panel.grid.minor.y = element_blank()) +
        labs(x = "in-sample census tracts",
             y = "",
             size = " ",
             color = " ") +
        facet_wrap(~version, ncol = 1) +
        scale_size_continuous(limits = c(0, 40), breaks = c(2, 6, 8, 10, 20, 30)) +
        scale_color_continuous(limits = c(0, 40), breaks = c(2, 6, 8, 10, 20, 30)) +
        guides(color = guide_legend(), size = guide_legend()) +
        scale_y_continuous(breaks = seq(2009, 2016, 1)) +
        scale_x_continuous(breaks = seq(1, 161, 40))
}
ggsave("samplesizes.png", 
       plot = fig2(ydat),
       device = "png",
       path = figures4paper.dir,
       width = 8, height = 4,
       units = "in",
       dpi = 600,
       bg = "white")
```

Effective sample size for paper:

```
ydat %>% balance_individual_data(sum.times = 3, sum.places = 1) %>%
    pull(s.wt) %>% sum()
#> [1] 1167
```

Let’s run the repetitions.

```
py.normed <- inner_join(pdat, ydat, by = c("place", "time")) %>%
    norm_individual_cell.weights()

places.in.sample <- unique(py.normed$place)

num.reps <- 10

set.seed(456)
s.seeds <- sample(.Machine$integer.max, size = num.reps)
z.seeds <- sample(.Machine$integer.max, size = length(places.in.sample))

loo.bal.reps <- list()

for (s in 1:num.reps) {
    
    s.seed <- s.seeds[s]
    
    set.seed(s.seed)
    s.py <- py.normed %>%
        sample_individual_data(num.times = 3, num.places = 1)
    
    s.rep <- list()
    s.rep$ypreds <- NULL
    s.rep$vi.loo  <- NULL
    
    # LOO (places-in-sample) part
    for (z in 1:length(places.in.sample)) {
        
        z.place <- places.in.sample[z]
        z.pdat <- subset(pdat, place==z.place) 
        
        zs.py <- s.py %>%  
            rm_place_and_connected_persons(place = z.place) %>%
            collect_binary.y()  
        
        z.seed <- z.seeds[z]
    
        zs.rf <- 
            grf..regression_forest(formula     = model.form,
                                   data        = zs.py,
                                   s.wt.var    = "s.wt",
                                   cluster.var = "place",
                                   
                                   ci.group.size = 1,
                                   sample.fraction = .7,
                                   compute.oob.predictions = FALSE,
                                   seed = z.seed)
        
        s.rep$ypreds <- get_ypred.oos(dat     = z.pdat,
                                        rf      = zs.rf,
                                        ypreds = s.rep$ypreds,
                                        loo     = TRUE)
        
        s.rep$vi.loo <- cbind(s.rep$vi.loo, grf..variable_importance(zs.rf))
        colnames(s.rep$vi.loo)[z] <- as.character(z.place)
        
        rm(z.place, z.pdat, zs.py, z.seed, zs.rf)
        
        if (z%%5==0) gc()
    }
    
    rm(z)
    
    s.rep$vi.loo.ave <- rowMeans(s.rep$vi.loo)
    
    
    # non-LOO (places-out-of-sample) part
    s.rf <- grf..regression_forest(formula     = model.form,
                                   data        = s.py,
                                   s.wt.var    = "s.wt",
                                   cluster.var = "place",
                                   
                                   ci.group.size = 1,
                                   sample.fraction = .7,
                                   compute.oob.predictions = FALSE,
                                   seed = s.seed)
    
    s.rep$ypreds <- get_ypred.oos(dat = pdat,
                                    rf = s.rf,
                                    ypreds = s.rep$ypreds)
    
    s.rep$vi.oos <- grf..variable_importance(s.rf)
    
    rm(s.py, s.seed, s.rf)
    
    s.rep$rmse <- compute_rmse(ydat = ydat, s.rep$ypreds,
                               balance.individuals = TRUE)

    
    loo.bal.reps[[s]] <- s.rep;  rm(s.rep)
    
    # just being careful, save result after each repetition 
    # (don't need this, as will save later)
    saveRDS(loo.bal.reps,
            here::here("vignettes", "results", "loo-bal.rds"))
}

rm(py.normed, places.in.sample, num.reps, s.seeds, s, z.seeds)
```

Now let’s pool the results from the repetitions.

```
loo.bal <- list()

# compute ypreds by averaging across repetitions
loo.bal$ypreds <- loo.bal.reps[[1]]$ypreds[c("place", "time")]
loo.bal$ypreds$ypred <- 
    rowMeans(
        sapply(1:length(loo.bal.reps), function(u) {
            loo.bal.reps[[u]]$ypreds$ypred
        })
    )

# compute RMSE on these ypreds
loo.bal$rmse <- compute_rmse(ydat = ydat, 
                             ypreds = loo.bal$ypreds, 
                             balance.individuals = TRUE)

# compute variable importance by averaging across repetitions
loo.bal$vi <- 
    cbind(loo = rowMeans(sapply(1:length(loo.bal.reps), 
                                function(s) {
                                    loo.bal.reps[[s]]$vi.loo.ave
                                })),
          oos = rowMeans(sapply(1:length(loo.bal.reps),
                                 function(s) {
                                     c(loo.bal.reps[[s]]$vi.oos)
                                 })))

# save pooled results together with the repetitions
saveRDS(list(loo.bal.reps = loo.bal.reps,
             loo.bal      = loo.bal),
        file = here::here("vignettes", "results", "loo-bal.rds"))
```

The variable importance metric tells us that the most important place
characteristics for predicting the outcome are `juvedrug`,
`domio` and `gunhom`.

```
p1 <- plot_vi(loo.bal$vi, "loo") + ggtitle("LOO part")
p2 <- plot_vi(loo.bal$vi, "oos") + ggtitle("OOS part")
gridExtra::grid.arrange(p1, p2, ncol = 2)
```

```
ggsave("variableimportance.png", 
       plot = gridExtra::grid.arrange(p1, p2, ncol = 2),
       device = "png",
       path = figures4paper.dir,
       width = 8, height = 4,
       units = "in",
       dpi = 600,
       bg = "white")
```

Let’s have a look at the predictions.

```
plot_ypreds(loo.bal$ypreds, type = "distributions")
```

```
plot_ypreds(loo.bal$ypreds, type = "values")
```

## Method issue 4: Some place variables are not available for all years

We have seen from an earlier section, restricting analysis to the
2009-2016 year range greatly standardized data availability, but some
variables are still not available for all the years in this range. Of
the three place variables with the largest variable importance,
`domvio` is available for only three of the eights years.
There’s nothing we can do about this except recognizing that V-score
estimation for those years benefit from more data. The other two
variables, `juvedrug` and `gunhom`, each are
available for all but one year – 2016 for `juvedrug` and 2009
for `gunhom`. A question is whether we can do better for
these two years. A reasonable idea is to bring in a proxy for the
unavailable variable if such a proxy variable is available. We need to
be cautious about the use of proxy variables, though, as they bring both
relevant information and noise. In the current case, the variable that
might serve as proxy is the same variable from an adjacent year. This
would introduce another layer of complexity, since the V-score estimated
would have slightly different meaning depending on the year, which may
complicate/limit its utility for users.

That said, let’s try this strategy. We run the whole procedure
described above twice, each time replacing one of the two variables
`juvedrug` and `gunhom` with its adjacent year
version. We then take the (raw) scores for 2016 scores from the first ad
hoc procedure and for 2009 from the second ad hoc procedure to replace
the scores for those years from our original results.

It is interesting that while this increased the variability of the
predictions for 2016 (standard deviation (SD) rose from 0.032 to 0.036)
and 2009 (SD rose from 0.062 to 0.066), it did not improve MSE and
actually slightly worsen it (from 0.4579 to 0.4583). Proxying is not
worth it.

## Method issue 5: Time trends in the outcome data

```
ydat %>% balance_individual_data(sum.times = 3) %>%
    group_by(time) %>% 
    summarize(y.mean = weighted.mean(y, s.wt),
              size = sum(s.wt))
#> # A tibble: 8 × 3
#>    time y.mean  size
#>   <dbl>  <dbl> <dbl>
#> 1  2009  0.531  164.
#> 2  2010  0.406  165.
#> 3  2011  0.324  161.
#> 4  2012  0.306  158.
#> 5  2013  0.282  132.
#> 6  2014  0.233  120.
#> 7  2015  0.227  118.
#> 8  2016  0.217  148.
```

Let’s standardize the predicted probabilities to construct the
V-scores.

```
vscores.year <- loo.bal$ypreds %>%
    group_by(time) %>%
    mutate(vscore = (ypred - mean(ypred)) / sd(ypred)) %>%
    ungroup()
```

## Consideration 6: average Vscore

Correlations among year-specific V-scores

```
vscores.cormat <- vscores.year %>%
    pivot_wider(names_from = time, names_prefix = "VS", id_cols = place, values_from = vscore) %>%
    select(-place) %>%
    as.matrix() %>%
    cor() %>%
    round(3)
vscores.cormat
#>        VS2009 VS2010 VS2011 VS2012 VS2013 VS2014 VS2015 VS2016
#> VS2009  1.000  0.901  0.874  0.866  0.853  0.818  0.808  0.767
#> VS2010  0.901  1.000  0.932  0.913  0.902  0.853  0.848  0.834
#> VS2011  0.874  0.932  1.000  0.924  0.897  0.849  0.832  0.831
#> VS2012  0.866  0.913  0.924  1.000  0.919  0.890  0.858  0.847
#> VS2013  0.853  0.902  0.897  0.919  1.000  0.910  0.874  0.864
#> VS2014  0.818  0.853  0.849  0.890  0.910  1.000  0.867  0.870
#> VS2015  0.808  0.848  0.832  0.858  0.874  0.867  1.000  0.885
#> VS2016  0.767  0.834  0.831  0.847  0.864  0.870  0.885  1.000

# average of adjacent year correlations
mean(sapply(2:nrow(vscores.cormat), function(z) {
    vscores.cormat[z, z-1]
}))
#> [1] 0.9054286
```

```
vscores.comb <- vscores.year %>%
    group_by(place) %>%
    summarize(vscore = mean(vscore)) %>%
    ungroup() %>%
    mutate(vscore = (vscore - mean(vscore)) / sd(vscore))
```

Let’s have a look at the final scores

```
plot_vscores(vscores.year, include.generic = TRUE, type = "values")
```

```
plot_vscores(vscores.year, include.generic = TRUE, type = "distributions")
```

Figure 4 for paper:

```
fig4 <- function(dat) {

    
    pdat <- dat %>% 
        gather(key = "type", value = "value", -c(place, time)) %>%
        mutate(generic = FALSE)
    
    avscores <- dat %>%
        group_by(place) %>%
        summarize(value = mean(vscore)) %>%
        ungroup() %>%
        mutate(value = (value - mean(value)) / sd(value)) %>%
        mutate(type = "vscore",
               time = max(dat$time) + 1,
               generic = TRUE)
    
    pdat <- bind_rows(pdat, avscores) %>%
        mutate(type = factor(type,
                             levels = c("ypred", "vscore"),
                             labels = c("predicted probabilities",
                                        "V-scores")))
    
    pdat$time.lab <- as.character(pdat$time)
    pdat$time.lab <- ifelse(pdat$time.lab==max(dat$time)+1,
                            "generic",
                            pdat$time.lab)
    
    pdat$generic <- (pdat$time.lab=="generic")

        time.labs <- pdat %>%
            select(time, time.lab) %>%
            distinct() %>%
            arrange(time)
    
    p <- pdat  %>%
        ggplot(aes(x = time, y = value, color = generic)) +
        geom_jitter(width = .25, height = 0, alpha = .3) +
        facet_grid(type~., scales = "free_y") +
        scale_x_continuous(breaks = time.labs$time,
                           labels = time.labs$time.lab) +
                scale_color_manual(values = c("black", "blue")) +
        labs(x = "", y = "") +
        theme_bw() +
        theme(legend.position = "none")

    return(p)
}

ggsave("preds_scores.png",
       plot = fig4(vscores.year),
       device = "png",
       path = figures4paper.dir,
       width = 8, height = 4,
       units = "in",
       dpi = 600)
```

Save estimated V-scores

```
saveRDS(vscores.year,
        file = here::here("data-raw", "vscores-ct", "vscore-year.rds"))
saveRDS(vscores.comb,
        file = here::here("data-raw", "vscores-ct", "vscore-comb.rds"))
```
